# Welcome to the big leaves: Best practices for improving genome annotation in non‐model plant genomes

**DOI:** 10.1002/aps3.11533

**Published:** 2023-08-08

**Authors:** Vidya S. Vuruputoor, Daniel Monyak, Karl C. Fetter, Cynthia Webster, Akriti Bhattarai, Bikash Shrestha, Sumaira Zaman, Jeremy Bennett, Susan L. McEvoy, Madison Caballero, Jill L. Wegrzyn

**Affiliations:** ^1^ Department of Ecology and Evolutionary Biology University of Connecticut Storrs Connecticut 06269 USA

**Keywords:** BRAKER, gene identification, genome annotation, MAKER, plant genomes, StringTie2, TSEBRA

## Abstract

**Premise:**

Robust standards to evaluate quality and completeness are lacking in eukaryotic structural genome annotation, as genome annotation software is developed using model organisms and typically lacks benchmarking to comprehensively evaluate the quality and accuracy of the final predictions. The annotation of plant genomes is particularly challenging due to their large sizes, abundant transposable elements, and variable ploidies. This study investigates the impact of genome quality, complexity, sequence read input, and method on protein‐coding gene predictions.

**Methods:**

The impact of repeat masking, long‐read and short‐read inputs, and de novo and genome‐guided protein evidence was examined in the context of the popular BRAKER and MAKER workflows for five plant genomes. The annotations were benchmarked for structural traits and sequence similarity.

**Results:**

Benchmarks that reflect gene structures, reciprocal similarity search alignments, and mono‐exonic/multi‐exonic gene counts provide a more complete view of annotation accuracy. Transcripts derived from RNA‐read alignments alone are not sufficient for genome annotation. Gene prediction workflows that combine evidence‐based and ab initio approaches are recommended, and a combination of short and long reads can improve genome annotation. Adding protein evidence from de novo assemblies, genome‐guided transcriptome assemblies, or full‐length proteins from OrthoDB generates more putative false positives as implemented in the current workflows. Post‐processing with functional and structural filters is highly recommended.

**Discussion:**

While the annotation of non‐model plant genomes remains complex, this study provides recommendations for inputs and methodological approaches. We discuss a set of best practices to generate an optimal plant genome annotation and present a more robust set of metrics to evaluate the resulting predictions.

The first published plant genome, *Arabidopsis thaliana* (L.) Heynh., was released in 2000 (Arabidopsis Genome Initiative, 2000). Its small size (135 Mbp) and minimal repeat content stand in stark contrast to the plant genomes sequenced and assembled today (Kress et al., 2022). The National Center for Biotechnology Information (NCBI; https://www.ncbi.nlm.nih.gov/) genome repository contains the genomes of over 900 land plant species, and roughly half of these are assembled to chromosome scale. The total number of complete reference plant genomes has more than doubled in the past five years (Marks et al., 2021). Initiatives like the Open Green Genomes (https://phytozome-next.jgi.doe.gov/ogg/), 10KP (Cheng et al., 2018), and the Earth BioGenome Project (Lewin et al., 2022) are improving the phylogenetic representation of plant genomes by sampling underrepresented clades. The plant genomes published today are more likely to be polyploids and/or larger genomes with substantial transposable element (TE) contents (Sun et al., 2022). Recent high‐throughput sequencing advancements, particularly long reads and chromosome conformation capture approaches, have enabled the completion of these more challenging assemblies (Pucker et al., 2022).

While genome assembly has seen substantial improvements in accuracy and contiguity, structural annotation remains challenging. This process delineates the physical positions of genomic features, including protein‐coding genes, promoters, and regulatory elements. It can be followed by functional annotation, which assigns biological descriptors to the identified features. The accurate classification of these features provides the basis for questions focused on species evolution, population dynamics, and functional genomics. Errors in genome annotation are frequent, even among well‐studied models, and are propagated through downstream analyses (Deutekom et al., 2019; Salzberg, 2019; Meyer et al., 2020). In most eukaryotes, genome annotation is made more challenging by the partial conservation of sequence patterns, variable intron lengths, variable distances between genes, alternative splicing, and higher densities of TEs and pseudogenes (Kersey, 2019; Salzberg, 2019). As a result of these complexities, the structural annotation process requires more advanced informatic tools and skills that support the integration and manipulation of large data sets (Mudge and Harrow, 2016).

Structural and functional genome annotation proceeds in three stages: identifying and masking noncoding regions (repeats), predicting the physical positions of gene structures, and assigning biological information to the predictions (Jung et al., 2020). Repeat regions are soft‐masked (e.g., RepeatMasker [Smit et al., 2013–2015] and RepeatModeler2 [Flynn et al., 2020]), which means these regions are indicated but not obscured to annotation software. This is followed by gene prediction, which may be ab initio (evidence‐free) or evidenced‐based. Evidence‐based approaches use RNA sequencing (RNA‐Seq) and protein sequence similarity search alignments. Evidence‐based approaches are often used in combination with ab initio approaches (e.g., AUGUSTUS [Stanke and Waack, 2003]) to generate models trained on patterns associated with true genes. Given the advanced state of high‐throughput transcriptome sequencing, it is common to resolve transcripts from RNA reads through genome‐guided approaches, such as using StringTie2 (Kovaka et al., 2019). Long‐read cDNA sequencing through Pacific Biosciences (PacBio) and Oxford Nanopore can provide additional resolution and improve the identification of splice variants. When extrinsic evidence from RNA‐Seq and protein alignments are available, workflow packages such as MAKER (Cantarel et al., 2008; Holt and Yandell, 2011; Campbell et al., 2014a) and BRAKER (Hoff et al., 2016, 2019; Brůna et al., 2021) can assist in training prediction tools ab initio. These packages can leverage sequence data from the target species, as well as external evidence from closely related species. While these workflows can simplify the integration across external evidence, downstream packages are still required to select or modify the resulting predictions (Haas et al., 2008; Banerjee et al., 2021; Gabriel et al., 2021).

Here, we provide a comprehensive evaluation of plant genome annotation workflows, intentionally selecting beyond the typical model species to represent some of the more complex genomes under investigation today. In doing so, we evaluate the impact of repeat masking using two different implementations of the RepeatModeler2 framework (Flynn et al., 2020). This is followed by exploring the role of read length and accuracy, and the impact of short‐read and long‐read data. Finally, we examine the contribution of protein evidence, generated from the de novo assembly of the RNA inputs and a genome‐guided assembly. These variations are examined in the MAKER and BRAKER frameworks to emphasize the importance of defining benchmarks to guide downstream filtering approaches. Finally, the largest and most repetitive genome assessed in this study, *Liriodendron chinense* (Hemsl.) Sarg., was used to demonstrate best practices to refine the predictions.

## METHODS

### Gathering plant genome data sets

Five plant genomes were chosen for this study, including the Chinese tulip tree (*Liriodendron chinense*) (Chen et al., 2019), black cottonwood (*Populus trichocarpa* Torr. & A. Gray ex Hook. version 3) (Tuskan et al., 2006), Chinese rose (*Rosa chinensis* Jacq.) (Raymond et al., 2018), thale cress (*Arabidopsis thaliana* TAIR 10) (Cheng et al., 2017), and a bryophyte, the common cord‐moss (*Funaria hygrometrica* Hedw.) (Kirbis et al., 2022) (Appendix S1). The genomes were selected to represent two model systems (*Populus* L. and *Arabidopsis* Heynh.) with well‐curated structural annotations and three non‐model systems, for which computational techniques were exclusively used to produce the annotations. Two of these non‐model systems were also more divergent examples, representing the only sequenced member of their genus (*Funaria* Hedw. and *Liriodendron* L.). The publicly available assembly and annotations for each species were accessed from NCBI, and genome completeness was estimated by searching the genome and annotation for the conserved single‐copy orthologs in the Embryophyta odb10 BUSCO version 5.0.0 (Simão et al., 2015). The contiguity of the reference genomes was assessed using Quast version 5.0.2 (Gurevich et al., 2013). The published annotation files were summarized using gFACs (Caballero and Wegrzyn, 2019).

Read sets available through NCBI's Sequence Read Archive (SRA) were accessed to provide transcriptomic evidence for each species and included a variety of tissue types. The Illumina short‐read libraries were sequenced (100‐bp paired‐end) with a HiSeq 2500 (Illumina, San Diego, California, USA). The read sets included at least four libraries, 20–82 million reads before the quality control (QC), and a minimum of 16 million reads after QC. Iso‐Seq (PacBio, Menlo Park, California, USA) long reads were accessed for *Populus* and *Liriodendron*, and PromethION (Oxford Nanopore Technologies [ONT], Oxford, United Kingdom) reads were available for *Rosa* and *Arabidopsis*. The read sets for long‐read data ranged from 161,000 to 41 million total reads per species (Appendix S2).

### Repeat masking and read alignment

RepeatModeler2 (Flynn et al., 2020) was used to construct repeat libraries with default settings, and the repeats were soft masked with the libraries constructed via RepeatMasker version 4.0.6 (Smit et al., 2013–2015). The genomes of *Arabidopsis*, *Funaria*, *Populus*, and *Liriodendron* were additionally masked using RepeatModeler2 with additional long terminal repeat (LTR) identification (‐LTRStruct flag). A quality assessment of the Illumina short reads was performed using FastQC version 0.11.7 (Andrews, 2010) before and after trimming the low‐quality bases. Sickle version 1.33 (Joshi and Fass, 2011) was used to trim low‐quality bases, with 50 bp as the minimum read length threshold. Single‐end reads generated post‐trimming were excluded from the RNA alignments and assembly. The trimmed short reads were aligned against their reference genomes using HISAT2 version 2.2.0 (Kim et al., 2019). HISAT2 was selected for its performance in recent benchmarking studies and as the aligner of choice for input to StringTie2 (Corchete et al., 2020; Musich et al., 2021). Long‐read RNA data were obtained for four species: *Arabidopsis* and *Rosa* were sequenced with ONT's PromethION, and *Populus* and *Liriodendron* were sequenced with PacBio Sequel. The long‐read data sets were aligned against their respective genomes using Minimap2 version 2.1.7 (Li, 2018, 2021).

### Generation of protein evidence

To generate protein evidence, Illumina short reads were assembled de novo using Trinity version 2.8.5 (Grabherr et al., 2011), with a minimum contig length of 300 bp. The assembled transcriptomes for the multiple libraries were combined, and putative coding regions were predicted using TransDecoder version 5.3.0 (http://transdecoder.github.io). TransDecoder is one of several frame‐selection methods available and performs in a comparable manner but is not always superior in all metrics (Bolger et al., 2018). For this study, it was selected as the most widely used package for this purpose. Redundancy in the predicted coding regions was reduced after clustering at 98% identity using UCLUST, a clustering algorithm of USEARCH version 9.0.2132 (Edgar, 2010). Frame‐selected transcripts shorter than 300 bp were removed. The remaining transcripts were aligned to the genome using GMAP version 2019‐06‐10 (Wu and Watanabe, 2005). The predicted proteins (from the same Transdecoder run) were aligned to the reference genome using GenomeThreader version 1.7.1 (Gremme et al., 2005).

To provide protein evidence from genome‐guided sources, the previously aligned Illumina short reads (via HISAT2) were constructed into transcripts with StringTie2 version 2.2.0 (Pertea et al., 2015; Kovaka et al., 2019). Long reads were treated similarly, along with a combination of short and long reads. The predicted transcripts were extracted using gffRead (Pertea and Pertea, 2020) and frame‐selected with TransDecoder. The transcriptome alignment annotation file (gff3) was passed to gFACs for the evaluation of the gene model statistics. The completeness of the aligned transcripts and protein sequences was estimated using BUSCO. Finally, to provide evidence from external sources (not derived from any transcriptomic inputs), full‐length protein evidence from OrthoDB (odb10_plants; Kriventseva et al., 2019) was provided for BRAKER/TSEBRA runs.

### Genome annotations

Each genome was tested in four primary open‐source annotation softwares to predict the gene models (Table 1). Several different runs of BRAKER version 2.1.5 (Hoff et al., 2019) and BRAKER/TSEBRA (Gabriel et al., 2021) were used with various combinations of RNA‐Seq (long‐ and short‐read inputs) and protein evidence. MAKER version 3.1.3 (Cantarel et al., 2008) was run once with the transcript and protein evidence. Finally, StringTie2 (Kovaka et al., 2019), with TransDecoder, was used to generate genome‐guided predictions from RNA evidence alone (Appendix S3).

**Table 1:** Notations for the different runs performed for benchmarking.

Run		*Arabidopsis*	*Funaria*	*Populus*	*Liriodendron*	*Rosa*
**StringTie2**						
**ST2 (SR)**	Short reads	X	X	X	X	X
**ST2 (LR)**	Long reads	X		X	X	X
**ST2 (SR/LR)**	Short and long reads	X		X	X	X
**BRAKER**						
**BR (SR)**	Short reads	X	X	X	X	X
**BR (LR)**	Long reads	X		X	X	X
**BR (SR/LR)**	Short and long reads	X		X	X	X
**BR (SR/RM2+)**	Short reads with additional masking for LTRs	X	X	X	X	
**TSEBRA**						
**TSB (SR/TRINITY)**	Short reads and de novo proteins	X	X	X	X	X
**TSB (SR/ST2)**	Short reads and genome‐guided proteins	X	X	X	X	X
**TSB (LR/ST2)**	Long reads and genome‐guided proteins	X		X	X	X
**TSB (SR/LR/ST2)**	Short and long reads and genome‐guided proteins	X		X	X	X
**TSB (SR/ST2/RM2+)**	Short reads and genome‐guided proteins with additional masking for LTRs	X	X	X	X	
**TSB (SR/OrthoDB)**	Short reads and OrthoDB proteins	X	X	X	X	X
**MAKER**						
**MK (RM2+)**	Short reads with additional masking for LTRs	X	X	X		

*Note*: LR = long reads; LTR = long terminal repeats; RM2+ = RepeatModeler2 with the additional repeat masking; SR = short reads.

### MAKER annotation

MAKER (MK) was run on the soft‐masked reference genomes of *Arabidopsis*, *Populus*, and *Funaria*, with repeats estimated using the additional LTR detection method in RepeatModeler2 (LTRStruct flag; RM2+). This was intended to emulate the MAKER‐P (Campbell et al., 2014b) method because the original repeat and pseudogene identification protocols are deprecated. MK (RM2+) was executed (i.e., trained) twice. The annotations derived from MK (RM2+) used protein evidence generated from de novo–assembled RNA reads from Trinity. These models were used to train the ab initio gene prediction software AUGUSTUS version 3.3.3 (Stanke and Waack, 2003) and SNAP version 2006‐07‐28 (Korf, 2004). The hidden Markov models (HMMs) trained using AUGUSTUS and SNAP were used, along with initial aligned evidence (est2genome and protein2genome parameters) for the second MK (RM2+) run to generate the final gene models.

### Assessment of gene predictions

The quality of genome annotations among the different gene prediction methods was evaluated using three primary metrics: (1) the mono‐exonic (single‐exon) and multi‐exonic (multiple‐exon) ratio, (2) conserved single‐copy orthologs queried from the predicted gene models using BUSCO (embryophyta database version 10), and (3) gene prediction assessment with EnTAP version 0.10.8 (Hart et al., 2020) using a 70% reciprocal functional annotation approach with NCBI's RefSeq Plant and UniProt databases. The mono:multi ratio was calculated from the gFACs summary report run with default parameters (Caballero and Wegrzyn, 2019). We regard a mono:multi ratio near 0.2 to be ideal and have further validated this with a larger set of model plant genomes (Appendix S4) (Jain et al., 2008). The gene prediction assessment was recorded as a percentage of sequence similarity hits to the total number of genes. The value of the reciprocal BLAST search will be dependent on the phylogenetic relationships of the target species. While higher annotation rates indicate better gene prediction abilities, species‐specific genes will always be missed (Armisén et al., 2008); therefore, a minimum annotation rate of 80% is a reasonable threshold to expect when dealing with most non‐models. Similarly, a higher BUSCO score indicates a better annotation, as BUSCO utilizes OrthoDB to form its conserved sets; the recommended target score is >95% for land plants (Simão et al., 2015; Manni et al., 2021).

The sensitivity and precision of the runs for *Arabidopsis* and *Populus* were assessed using Mikado version 2.3.2 (Venturini et al., 2018), by comparing the predicted gene models to the current reference annotations.

### Post‐processing filtering

The predicted gene models for *Liriodendron* were taken a step further to refine the genome annotation. Post‐process filtering was performed using gFACs and assessed for improvement using BUSCO completeness scores and annotation rates. The mono‐exonic and multi‐exonic genes predicted for *Liriodendron* were filtered for unique genes. The mono‐exonic genes were further filtered for the presence of protein domains using InterProScan version 5.35‐74.0 and Pfam (Quevillon et al., 2005; Jones et al., 2014). Multi‐exonic genes that did not have an EggNOG (Huerta‐Cepas et al., 2019) or a sequence similarity hit were removed, and the final annotation was assessed using gFACs and EnTAP.

## RESULTS

### Genome sizes, repeats, and published annotations

**Figure 1:**
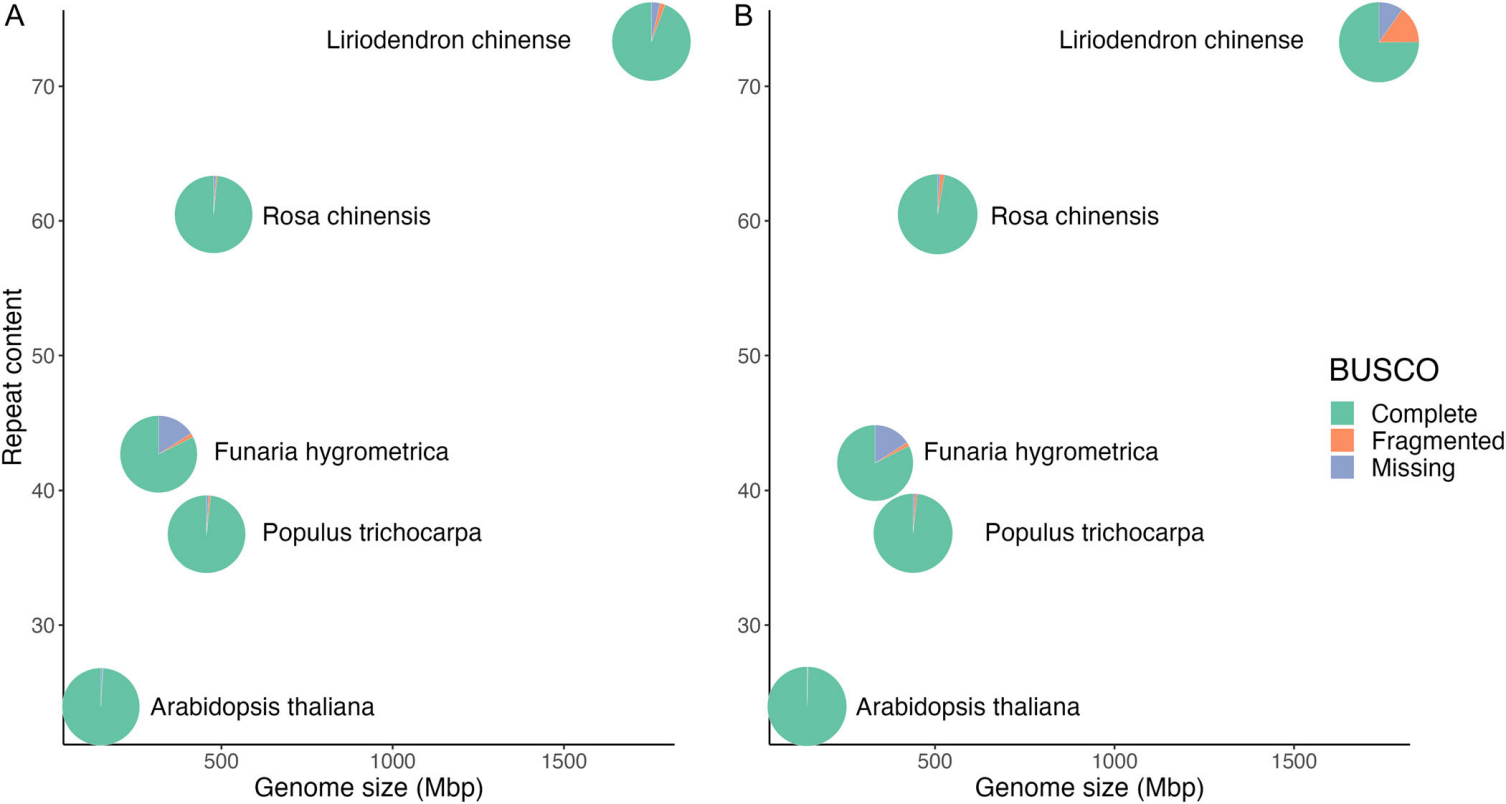
Genome size, repeat content, and BUSCO completeness for the five plant genomes: *Arabidopsis thaliana*, *Populus trichocarpa*, *Funaria hygrometrica*, *Rosa chinensis*, and *Liriodendron chinense*. Each pie represents the BUSCO completeness. Green denotes the completeness score, orange indicates the fragmented score, and blue indicates the missing score from BUSCO. (A) BUSCO scores estimated from the published assemblies. (B) BUSCO scores estimated from protein‐coding gene predictions from the published annotations.

The genome sizes of the five species assessed represented a 10‐fold difference between the smallest (*Arabidopsis* ~119 Mbp) and the largest (*Liriodendron* ~1.7 Gbp) (Figure 1A, Table 2). *Liriodendron* and *Rosa* have higher levels of repeat content (73.18% and 60.58%, respectively), and *Arabidopsis* has the lowest (23.9%). *Arabidopsis* is the most complete chromosome‐scale genome, with seven contigs reflecting its five chromosomes and two organellar chromosomes. The other genomes are assembled into pseudochromosomes (except for *Liriodendron*). Once the genomes were downloaded, contigs <500 bp were removed. The published genome assemblies and annotations were compared in terms of completeness using BUSCO (Figure 1, Table 2). When BUSCO is run in genome mode, it searches the genome for a set of 1614 single‐copy orthologs in the embryophyte database. Aside from *Funaria*, which had the lowest completeness score of 82.4%, the remaining plant genomes ranged from 94% to 99% complete. When we evaluated the published annotations for the same species and ran BUSCO in protein mode, a slightly lower level of completeness was observed in every species except *Funaria* and *Arabidopsis* (Figure 1B). The largest reduction in BUSCO score was observed in *Liriodendron* (98.6% vs. 75.1%). The discrepancy between the estimated completeness at the genome‐level and most of the published annotations speaks to the challenges of achieving an accurate structural annotation.

**Table 2:** Genome assembly and annotation statistics for the five published plant genomes.

Species	Genome size (Mbp)	Total scaffolds (chromosomes)	N50 (Mbp)	Repeat content	BUSCO completeness	
					Genome	Annotation
*Arabidopsis thaliana*	119	7 (5)	23.46	23.6%	99.30%	99.60%
*Funaria hygrometrica*	327	687 (26)	1.48	42.35%	85.60%	86.60%
*Liriodendron chinense*	1742	3711 (21)	3.53	73.18%	98.60%	75.10%
*Populus trichocarpa*	434	1446 (19)	19.47	35.90%	98.80%	98.30%
*Rosa chinensis*	515	55 (7)	69.64	60.53%	98.80%	97.30%

RepeatModeler2 with and without the LTRStruct package (the additional LTR masking module) (Flynn et al., 2020) was used to soft‐mask the repeats in four of the genomes. The increase in repeat content was marginal in all species, ranging from 1% in *Funaria* to 5% in *Populus*. Comparisons using the LTRStruct flag were denoted as RM2+ (Appendix S5).

### Transcriptome evidence

For the subsequent genome annotation analysis, the Illumina RNA short reads were first aligned to the genome. All libraries, ranging from four to 20 per species, aligned at over 97%, apart from *Rosa* (92%) (Table 3, Appendix S6). Long‐read RNA libraries were aligned with Minimap2 for four species: *Arabidopsis* (ONT PromethION reads, 97.1% aligned), *Populus* (PacBio Iso‐Seq reads, 92.01% aligned), *Liriodendron* (PacBio Iso‐Seq reads, 95.5% aligned), and *Rosa* (ONT PromethION reads, 99% aligned). The N50s for the long reads range from 976 bp in *Rosa* to 4.6 kbp in *Liriodendron* (Appendix S7).

**Table 3:** Comparison between genome‐guided (StringTie2) and de novo (Trinity) genome annotations.

Species	RM%	RM2 + %	Total short reads (total libraries)	Total long reads (total libraries)	Total Trinity transcripts (N50)	Total ST2 transcripts (SR) (N50)	Total ST2 transcripts (LR) (N50)	Total ST2 transcripts (SR/LR) (N50)	BUSCO transcript alignments (%)		
									Trinity (SR)	ST2 (SR)	ST2 (SR/LR)
*Arabidopsis*	15.2	16.5	511,277,126 (9)	23,134,068 (4)	319,434 (2726)	37,747 (2538)	36,241 (1599)	42,265 (1363)	82.7	95.5	93.6
*Funaria*	42.3	43.1	549,205,030 (9)		151,265 (1198)	59,741 (369)			72.9	84.5	
*Liriodendron*	73.2	72.7	1,408,831,670 (20)	10,437,029 (1)	2,839,867 (3055)	62,341 (1041)	33,895 (1815)	45,785 (2361)	92.5	87.1	77.3
*Populus*	35.9	45.1	267,403,772 (5)	161,334 (1)	283,572 (1837)	56,468 (402)	20,633 (1074)	37,222 (1869)	71.3	73.3	65.3
*Rosa*	60.5		134,461,068 (4)	41,929,383 (6)	812,407 (2187)	53,708 (672)	74397 (1866)	105,639 (1605)	88.8	97	97.2

*Note*: LR = long reads; RM = RepeatModeler2; RM2+= RepeatModeler2 with the LTRStruct flag; RM% = repeat content masked with RepeatModeler2; RM2 + % = repeat content masked with RepeatModeler2 with the LTRStruct flag; SR = short reads; ST2 = StringTie2.

### Transcript‐derived annotations

The reads were assembled using StringTie2 (ST2) and Trinity. The Trinity de novo assemblies of the Illumina short reads generated longer transcripts, with N50s ranging from 1.2 kbp (151,265 transcripts in total) in *Funaria* to 3.06 kbp (2,839,867 transcripts) in *Liriodendron*. Among the genome‐guided assemblies with StringTie2 (ST2 (SR)), the range was much smaller, with N50s ranging from 369 bp (59,741 transcripts in total) in *Funaria* to 2.54 kbp (37,747 transcripts) in *Arabidopsis* (Appendix S7). The StringTie2 (ST2 (LR) and ST2 (SR/LR)) range was longer, with N50s ranging from 1.07 kbp (20,633 transcripts in total) in *Rosa* ST2 (LR) to 2.36 kbp (45,785 transcripts) in *Liriodendron* ST2 (SR/LR) (Table 3, Appendix S7). The StringTie2 and Trinity transcripts were aligned back to the genome using GMAP after the frame selection. The BUSCO scores for the aligned transcriptomes derived from short‐read data, run in transcriptome mode, ranged from 73% in *Funaria* to 83% in *Rosa* for Trinity, and 73% in *Liriodendron* to 97% in *Rosa* using StringTie2 (Table 3). The BUSCO scores were the lowest for the ST2 (LR) runs across all species, as compared to the other StringTie2‐only runs. For ST2 (SR/LR), the BUSCO scores were lower than for ST2 (SR), except for *Rosa*, where the ST2 (SR/LR) was 97.2% as opposed to 97.0% in ST2 (SR). In all species, ST2 (SR/LR) had higher BUSCO scores than ST2 (LR). Despite Trinity producing more than double the total transcripts than StringTie2, the BUSCO completeness scores of most StringTie2 runs were much higher than that of Trinity. *Liriodendron* remained the only exception, with a slightly higher BUSCO score from Trinity.

**Figure 2:**
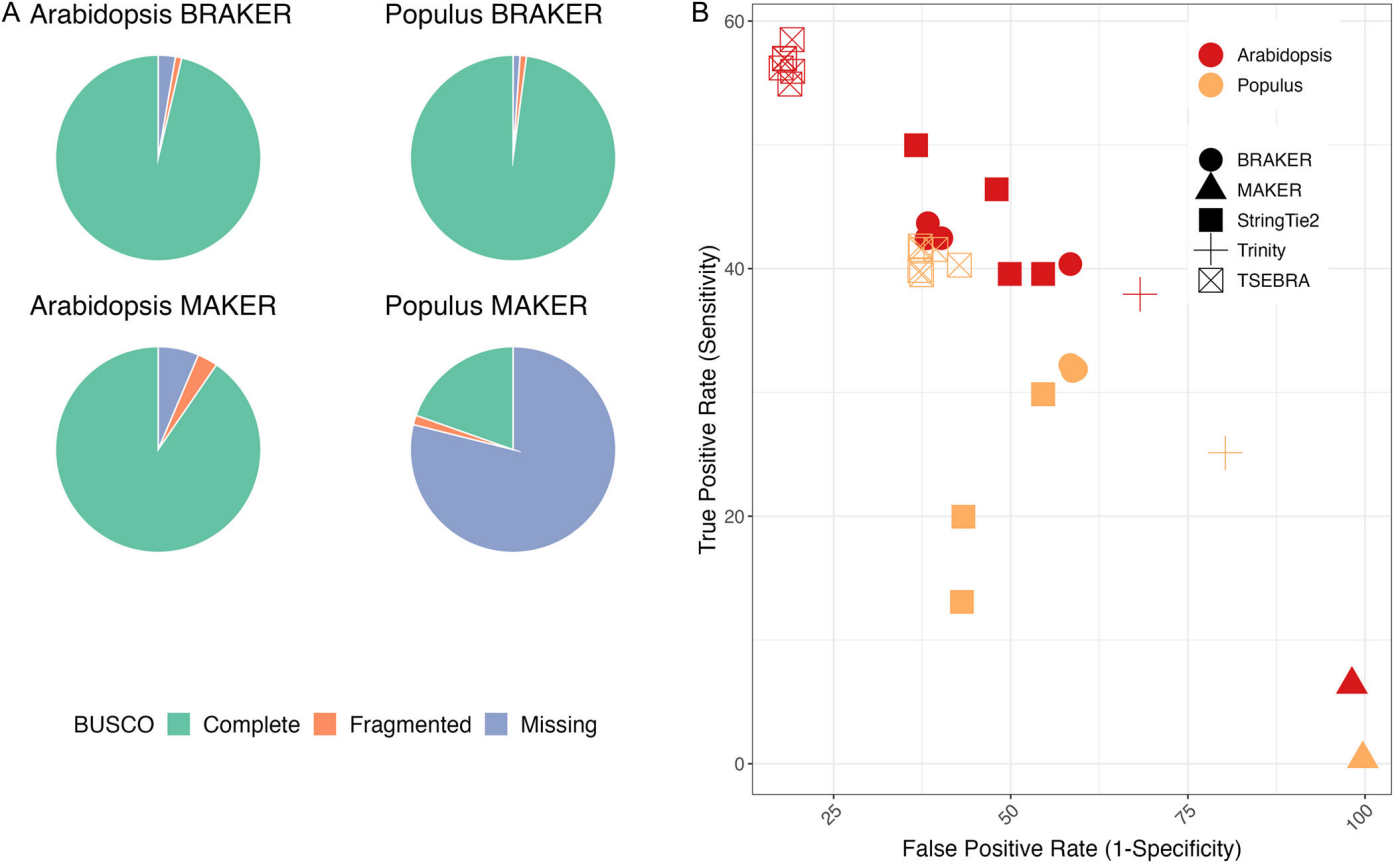
Comparison of BUSCO, sensitivity, and false positive rates between the *Arabidopsis* and *Populus* annotations (Appendix S8). (A) BUSCO completeness scores for the MK (SR/RM2+) and BR (SR/RM2+) runs of *Arabidopsis* and *Populus*. Green denotes the completeness score, orange indicates the fragmented score, and blue indicates the missing score from BUSCO. (B) False positive rates and sensitivity scores from Mikado against published annotations for *Arabidopsis* (red) and *Populus* (gold) for the MAKER, BRAKER, TSEBRA, Trinity, and StringTie2 runs. The scores were assessed using Mikado. Multiple points per run reflect differences in input read type and repeat masking.

*Arabidopsis* and *Populus* were further evaluated with Mikado to compare the sensitivity and specificity of the published annotations (Figure 2B, Appendix S8). Overall, the StringTie2 predictions had higher sensitivity and precision rates than the Trinity runs. From this point, Trinity was excluded, and StringTie2 runs were compared against the BRAKER and TSEBRA predictions (Figure 2B).

**Figure 3:**
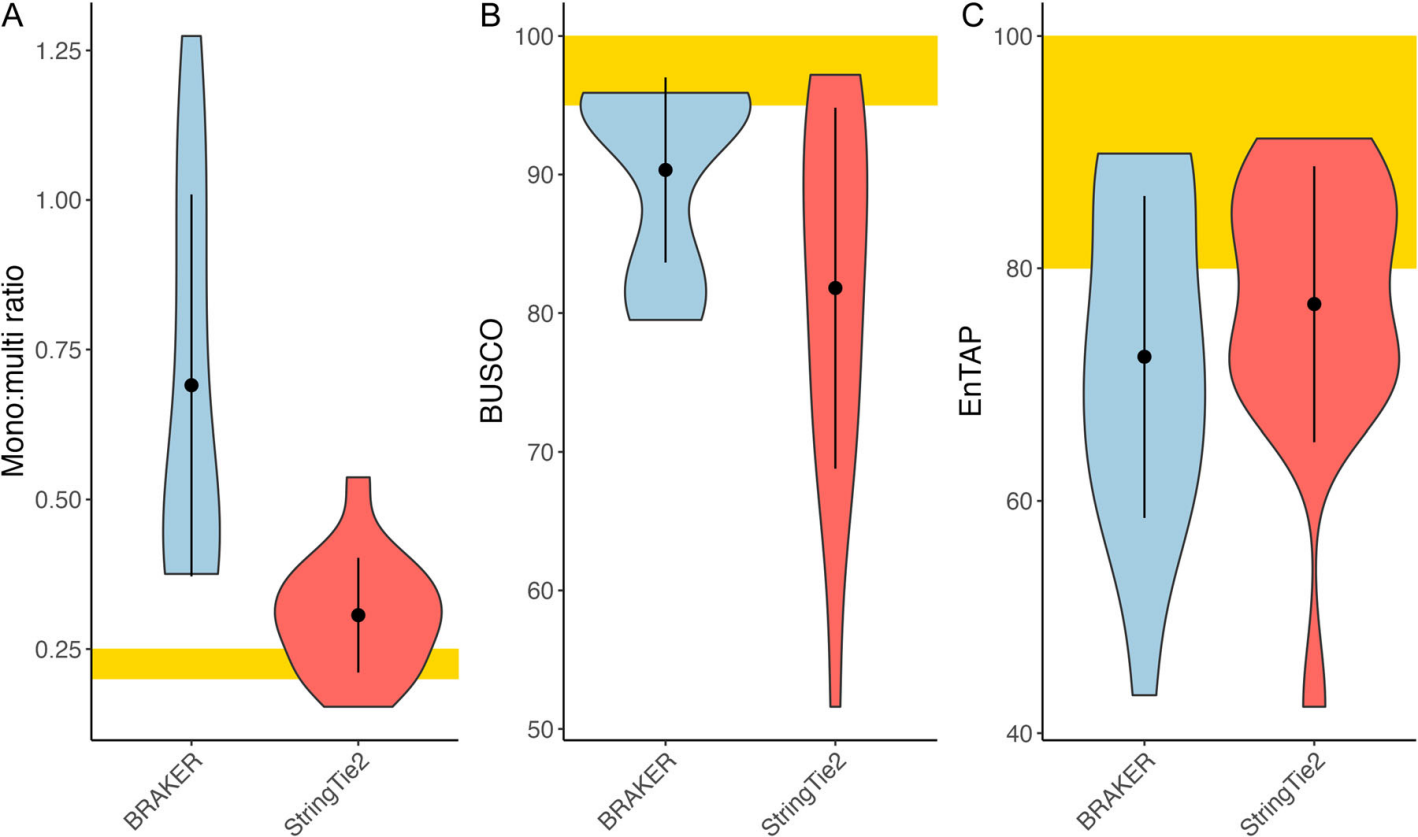
Comparing metrics between BRAKER (blue) and StringTie2 (red) predictions. (A) Mono:multi ratios, (B) BUSCO comparisons, and (C) EnTAP annotation rates of the gene models. The yellow region indicates the ideal value for each of the metrics.

The mono:multi ratios produced by StringTie2 ranged from 0.15 in *Populus* (ST2 (LR)) to 0.53 in *Liriodendron* (ST2 (LR)), which were an improvement over the mono:multi ratios produced from the BRAKER annotations that ranged from 0.37 in *Arabidopsis* (BR (LR)) to 1.27 in *Funaria* (BR (SR/RM2+)). The BUSCO scores of the proteins predicted from BRAKER were generally higher than the BUSCO scores from StringTie2; for example, *Arabidopsis* StringTie2 runs ranged from 85% (ST2 (LR)) to 95.5% in ST2 (SR), while BRAKER runs ranged from 94% (BR (LR)) to 95.9% (BR (SR)). Some runs are comparable, however; the ST2 (SR) run, BR (SR) run, and the BR (SR/RM2+) run in *Arabidopsis* all had a BUSCO score of 95%. StringTie2‐predicted models had a higher annotation rate, in general, than BRAKER; for example, the EnTAP annotation rate in *Funaria* was just over 40% post‐BRAKER but was near 60% from the StringTie2 runs (Figure 3).

### Genome annotation with MAKER

**Figure 4:**
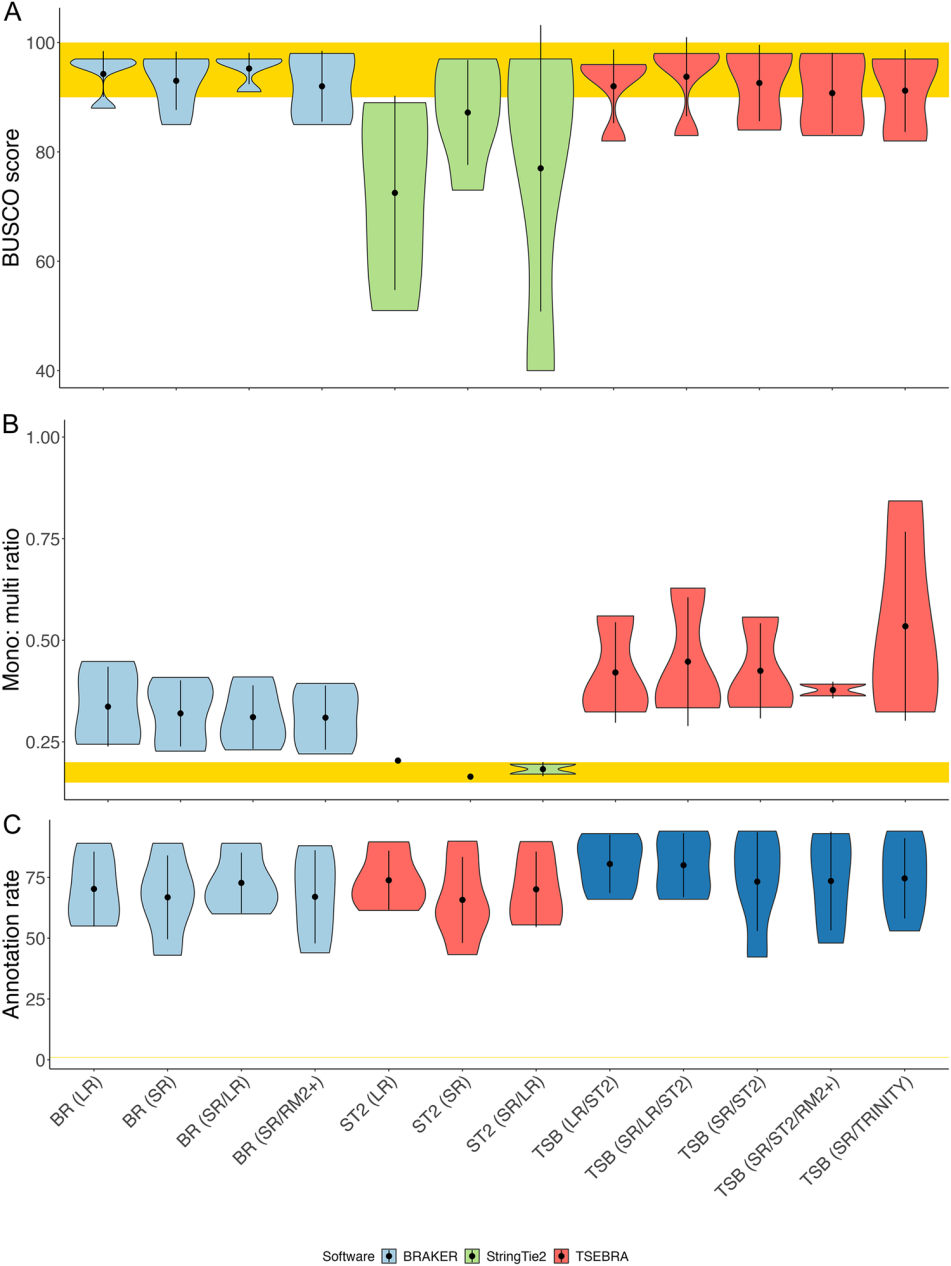
Comparison of scores across all species between the runs of different input types and software. (A) BUSCO completeness scores. (B) Mono:multi ratios. (C) EnTAP annotation rates. MAKER is shown in green, BRAKER is light blue, TSEBRA is dark blue, and StringTie2 is red. The yellow rectangle represents the target scores for each benchmark. RM2+, RepeatModeler2 with LTRStruct.

To replicate the repeat pipeline of MAKER‐P, the RM2+ genome was used for the MAKER runs for *Arabidopsis*, *Populus*, and *Funaria*. BUSCO completeness was low compared with the BRAKER runs, ranging from 19.6% in *Populus* to 90.4% in *Arabidopsis* (Figure 4A). The mono:multi ratio of MAKER(RM2+) for *Arabidopsis* was comparable to the BRAKER runs for the same species (0.22 for BR (SR) and BR (SR/RM2+), 0.24 for BR (LR), and 0.23 for BR (SR/LR)). The MK (RM2+) predictions for the total number of genes in *Arabidopsis* (22,000) and *Funaria* (44,000) were in the expected range for these species, whereas only 7000 genes were predicted for *Populus*. The average gene lengths ranged from 1.8 kbp in *Funaria* to 2.3 kbp in *Arabidopsis* (Appendix S9). The best MK (RM2+) run was for *Arabidopsis*, with a mono:multi ratio of 0.22 and a BUSCO score of 90.4%. On the other hand, the mono:multi ratio for *Populus* was 0.07, and the BUSCO score was 19.6%.

The model systems, *Arabidopsis* and *Populus*, were further evaluated with Mikado to compare the sensitivity and specificity of the published annotations (Figure 2B, Appendix S8). The sensitivity and precision scores for gene predictions were lowest from MAKER, followed by Trinity, and were highest from TSEBRA. StringTie2 and BRAKER yielded similar sensitivity and specificity scores for *Arabidopsis*, whereas for *Populus* the sensitivity score was lower than those from the BRAKER runs. Given its overall low scores, MAKER was excluded from the subsequent comparisons. It should be noted, however, that some protocols recommend using GeneMark in MAKER (Brůna et al., 2021). With this addition to the training protocol, MAKER was shown to have a higher accuracy in prediction.

### Genome annotation with BRAKER

In general, the BUSCO scores were improved in the BRAKER and TSEBRA runs, with the TSB (SR/OrthoDB) predictions scoring the highest. The best BUSCO scores were from TSB (SR/OrthoDB), ranging from 98.9% (TSB; SR/OrthoDB) in *Arabidopsis* to 82.2% in *Funaria*. Overall, the TSEBRA runs fared better, with the annotation completeness rates ranging from 99% in *Arabidopsis* (TSB; SR/OrthoDB) to 53% in *Funaria*. On the other hand, the TSEBRA runs had the worst mono:multi ratios; for example, the highest TSB (SR/ST2/RM2+) ratio was 1.27 for *Funaria* (Figure 4).

Overall, the gene models generated for *Arabidopsis* by BRAKER performed similarly across runs when evaluated using BUSCO scores. The mono:multi ratios across BRAKER runs ranged from 0.23 to 0.39, and the annotation rates were consistently above 95%.

The annotation rates for *Funaria* were lower than expected: 43% for BR (SR) and 53% for nearly all methods that included protein evidence (TSB (SR/TRINITY) and TSB (SR/OrthoDB)). The BUSCO completeness scores of about 85% post‐BRAKER are comparable to those from StringTie2.

In the case of the *Liriodendron* BRAKER and TSEBRA runs, the mono:multi ratios were more variable when compared to the StringTie2 runs, which ranged from 0.34 BR (SR) to 1.04 with BR (SR/RM2+). The annotation rates for each run were around 75%, with BUSCO scores from 83% with TSB (SR/LR/ST2) to 90.8% for BR (SR). *Populus* gene models post‐BRAKER without protein evidence had mono:multi scores around 0.24, and with TSEBRA, the ratio ranged from 0.4 to 0.5. The annotation rates also differed between BR (SR) (75%) and TSB (SR/ST2) (88%). *Rosa* had overall consistent scores (around 96%) for BUSCO post‐BRAKER. TSEBRA runs had higher mono:multi ratios (0.75) compared with the BRAKER runs (0.37) (Appendix S9).

### Annotation with long reads

For BRAKER runs, the predicted gene lengths from the long reads were comparable with those based on short reads, except for some runs with long reads within *Populus*. The average gene length for BRAKER with long reads for *Populus* ranged from 2.7 kbp to 3.4 kbp, although some transcripts exceed 6 kbp. The longest average gene lengths were observed in *Liriodendron* (9.3 kbp; BR (SR/LR)). The inclusion of long reads (exclusively) did not improve BUSCO completeness for any species, apart from *Arabidopsis*, where the BR (LR) BUSCO completeness was 1% higher than for the BR (SR) run. The increase in BUSCO completeness in *Arabidopsis* could be due to the large number of long reads included (23 million across four libraries); however, the quality of genome annotation does not seem correlated with the depth of long‐read sequencing. For example, *Rosa* had more reads (41 million across six libraries), and the BR (LR) run had a similar BUSCO score to BR (SR) (96%). It should be noted that the long reads for *Arabidopsis* and *Rosa* were sequenced with ONT PromethION. The PromethION reads had higher mapping rates, compared with Iso‐Seq, to their respective genomes: 97.1% in *Arabidopsis* and 99% in *Rosa* (Appendix S6). The long‐read inputs, regardless of depth or type, lowered the BUSCO completeness (up to 10%) across all ST2 (LR) runs (Appendix S10). Finally, we note that the combination of short reads and long reads (BR; SR/LR and TSB; SR/LR/ST2) is comparable to the BR (SR) reads in terms of BUSCO completeness. Exceptions to this case are *Arabidopsis* and *Rosa*, which had marginally higher BUSCO completeness scores. BR (SR/LR) runs produced more genes (in total) for *Arabidopsis*, *Populus*, and *Rosa* than BR (SR); however, fewer genes were identified in the TSB (SR/LR/ST2) runs than for BR (SR), apart from in *Rosa*. The annotation rate in the BR (SR/LR) of *Liriodendron* was higher than its BR (SR). The combination of proteins with SR and LR (TSB (SR/LR/ST2)) resulted in higher annotation rates across all species. The combination of SR and LR increased the mono:multi ratios, which were therefore worse across all species.

### Refining the genome annotation for *Liriodendron*

The BRAKER runs for *Liriodendron* were filtered with gFACs and InterProScan to remove unlikely gene models (Table 4). The number of mono‐exonic genes was drastically reduced post‐filter with InterProScan. Across all runs, the mono‐exonic genes numbered 11,000 to 25,000. After removing mono‐exonics without a protein domain annotation from the Pfam database, they decreased from 13,000 to 5000. The decrease in false positive mono‐exonics resulted in an improved mono:multi ratio that ranged from 0.16 for BR (SR) and BR (SR/RM2+), 0.16 and 0.23 for the StringTie2 runs, to 0.43 for the TSEBRA runs. The BUSCO scores decreased slightly post‐filtering (1–2%). EnTAP annotation rates ranged from 66% to 86%, with the TSB (SR/OrthoDB), which is an improvement from 59% to 68% pre‐filtering.

**Table 4:** Gene model statistics for *Liriodendron* after two rounds of structural and functional filters.

*Liriodendron* annotation^a^	Total genes	Mono:multi ratio	BUSCO %	EnTAP %
Published annotation	35,261	0.7	75.1	63
Mono‐exonic filters				
BR (LR)	39,031	0.21	87.4	69
BR (SR) *	41,065	0.16	90.2	66
BR (SR/LR) *	40,420	0.16	90.3	67
BR (SR/RM2+)	40,740	0.17	88.2	67
ST2 (SR)	51,804	0.16	86.5	80
ST2 (LR)	27,012	0.23	65	84
ST2 (SR/LR)	36,345	0.24	70.6	82
TSB (LR/ST2)	33,132	0.43	82.3	84
TSB (SR/LR/ST2)	33,964	0.43	82.4	84
TSB (SR/ST)	32,898	0.41	83.4	84
TSB (SR/ST2/RM2+)	33,637	0.45	82.8	84
TSB (SR/TRINITY)	34,646	0.42	84	83
TSB (SR/OrthoDB)	33,667	0.43	86.5	86
+Multi‐exonic filters				
BR (SR)	30,219	0.24	90.3	81
BR (SR/LR)	30,035	0.23	86.9	87

*Note*: BR = BRAKER; BUSCO % = completeness percentage; EnTAP % = annotation rates after reciprocal BLAST; LR = long reads; RM2+ = RepeatModeler2 with LTRStruct; SR = short reads; ST2 = StringTie2; TSB = TSEBRA.

^a^ Asterisks denote the two best annotation sets.

In terms of BUSCO completeness and mono:multi ratios, the two best‐performing runs (BR (SR) and BR (SR/LR)) were further filtered (Table 4). In this step, multi‐exonic genes without an EggNOG alignment or a sequence similarity alignment through EnTAP were removed. These filtered models were re‐assessed for their mono:multi ratio, BUSCO completeness, and EnTAP annotations. The BUSCO completeness remained the same for BR (SR), but not for BR (SR/LR). The EnTAP annotation increased from 66% to 81% in BR (SR), and 67% to 87% in BR (SR/LR).

## DISCUSSION

BRAKER (Hoff et al., 2019; Brůna et al., 2021) and MAKER (Cantarel et al., 2008) are currently the most popular eukaryotic structural annotation tools, cited 475 and 1010 times, respectively, since 2021, as referenced in Google Scholar (https://scholar.google.com/). Processes that select from multiple ab initio or aligned forms of evidence are gaining popularity as well, although they add both time and complexity to the analyses (FINDER cited 22 times [Banerjee et al., 2021]; EVidenceModeler cited 381 times [Haas et al., 2008]). Finally, as high‐throughput transcriptomics, in the form of both short‐ and long‐read evidence, become more accessible, rapid approaches such as StringTie2 (cited 451 times [Kovaka et al., 2019]) are occasionally used as the exclusive approach, although they are more often used in combination with the options listed above.

Regardless of the methods selected, recently published benchmarks demonstrate the challenge of achieving high values for gene sensitivity in larger genomes (Brůna et al., 2021). Within smaller and less complex model systems such as *Caenorhabditis elegans* and *Drosophila melanogaster*, the ab initio prediction results in gene sensitivities of 49.8% and 59.5%, respectively (Brůna et al., 2021). In well‐studied complex organisms, such as humans, the gene‐level sensitivity and specificity hovers at 48% and 43%, respectively (Banerjee et al., 2021). While generating benchmarks with model systems (*A. thaliana*, *C. elegans*, and *D. melanogaster*) provides more reliable metrics for comparison, they do not fully represent the diversity of their respective clades (Chang et al., 2016).

This study focused on four gene prediction workflows, StringTie2, MAKER, BRAKER, and BRAKER/TSEBRA, and examined the process across a variety of evidence inputs. Both model and non‐model plant genomes were considered to highlight the challenges and reinforce the need for downstream filtering.

### Genome annotation benchmarks for both models and non‐models

Among plant genomes, the total number of genes is relatively conserved and ranges from 20,000 to just over 40,000. As such, the total gene number provides an accessible preliminary benchmark; however, the number of genes in the reference annotation cannot be used to assess the overall quality of the annotation. To measure this, we should consider additional metrics. Here, we describe the utility of the BUSCO score, mono:multi‐exonic ratio, and sequence similarity assessment.

BUSCO allows us to identify complete, duplicated, fragmented, and missing single‐copy orthologs shared by most seed plants (Simão et al., 2015; Seppey et al., 2019). This provides a reliable benchmark in the absence of a high‐quality reference annotation. Poor BUSCO scores are immediately indicative of a larger issue; however, a high BUSCO score is not sufficient to estimate the quality of an annotation (Figure 4B). Six of the 18 BRAKER runs and four of the 17 StringTie2 runs exceeded 95% completeness, but their total gene number, gene length, and structure varied considerably.

Repeat content, especially in the form of LTRs, and pseudogenes can lead to inflated gene model estimates, especially for mono‐exonic genes (Scott et al., 2020; Trouern‐Trend et al., 2020). We expect that eukaryotes maintain 20% or less of their gene space as mono‐exonics (Jain et al., 2008; Appendix S4). Although the BUSCO scores were consistent, we note tremendous variation in the mono‐ to multi‐gene model ratios post‐BRAKER. In practice, having a worse mono:multi ratio is preferable to having a lower BUSCO score, as missing genes, especially those thought to be conserved, cannot be easily rectified, while putative false positives may be filtered out through other means.

Sequence similarity search metrics are more complex to interpret, but can provide a benchmark when used with high‐quality and curated databases that contain full‐length proteins (e.g., NCBI RefSeq). Specifically, a reciprocal BLAST search requires that both the query and target in the search retain a minimum level of coverage in the alignment. For new plant genomes in the darkest branches of the tree of life, this might be a less reliable metric. Some species may fare poorly in database comparisons, so searches for protein domains could provide some level of confidence. We demonstrate this as a filter to reduce the mono‐exonics in *Liriodendron* (Table 4).

### Repeat masking is important but may not require additional LTR resolution to improve performance

**Figure 5:**
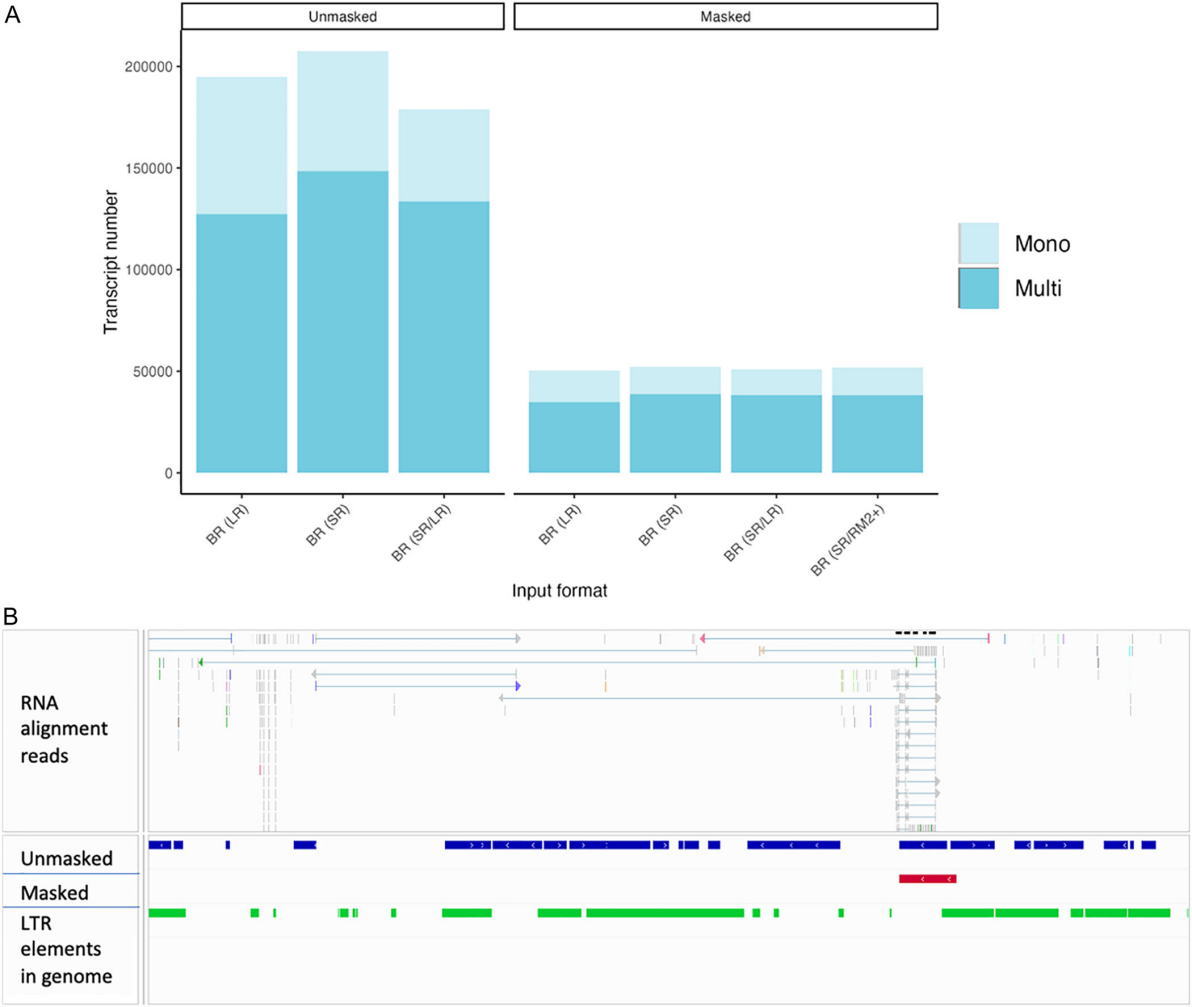
The effect of soft masking on gene prediction in *Liriodendron* (Appendix S11). (A) Performing structural annotation on the unmasked *Liriodendron* genome results in the identification of more mono‐exonic genes as opposed to multi‐exonic genes. Blue denotes the BRAKER (BR) runs for both genomes, SR denotes short reads, and LR denotes long reads. The lighter shade represents mono‐exonics, and the darker shade represents the multi‐exonics. (B) More genes predicted using the unmasked genome (blue), as compared with only one gene predicted in this region with the masked genome (red). The green track shows the long terminal repeat elements in the genome as identified by RepeatModeler2. The RNA alignment reads show a read pile‐up at the predicted gene (masked track).

Plant genomes typically contain many repeats, mostly in the form of TEs, averaging around 46% of the genome (Luo et al., 2022). Given the abundance of TEs in genomes, it is important to mask these in advance of gene prediction. Soft masking involves changing nucleotides identified as repeats to lowercase letters (Yandell and Ence, 2012), signaling downstream programs to ignore these sequences. Of the five genomes included in this study, *Liriodendron* had the largest genome size and highest repeat content. Running downstream analyses on an unmasked genome of *Liriodendron* resulted in a four‐fold increase in gene predictions (Figure 5A, Appendix S11). Many repeats were identified as putative gene models, resulting in a large increase in the total number of genes (Figure 5B, Appendix S11).

RepeatModeler2 is a widely used tool for TE discovery (Flynn et al., 2020). The recent release of RepeatModeler2 includes an optional module for more robust LTR structural detection (LTRStruct module) that includes the LTRharvest (Ellinghaus et al., 2008), LTRDetector (Valencia and Girgis, 2019), and LTR_retriever packages (Ou and Jiang, 2018). This is particularly useful in identifying more divergent LTRs in the genome that may exist in fewer copies (Ou and Jiang, 2018; Valencia and Girgis, 2019). Among the default packages included, RepeatScout serves as a fast method for detecting young and abundant repeat families in the genome. RECON, on the other hand, is more computationally intensive and is sensitive enough to detect older TE families. The LTRStruct module is run on the unmasked genome to identify LTR families that may be redundant with the families identified by the default package; this creates redundancy that is resolved through clustering with CD‐HIT (Flynn et al., 2020).

In the four species compared, additional repeat masking did not significantly improve gene predictions (Figure 4, Appendix S10). The mono:multi ratios across species were consistent before and after additional LTR masking (Figure 5A). The BUSCO completeness scores remained relatively the same, with BR (SR/RM2+) being 1% higher than BR (SR) in *Arabidopsis*, *Funaria*, and *Populus*. The marginal improvement observed in these genomes could be related to the structure and type of LTRs, such as the better identification of divergent Ty1‐copia elements described in the *Funaria* genome (Kirbis et al., 2022). Although we did not include genomes with excessive repeat estimates (>70%), our results indicated that the optional LTRStruct module was not beneficial.

### Transcripts derived directly from alignments are not sufficient to annotate reference genomes

Transcriptome assemblers are designed to work with primarily short RNA‐Seq reads to construct full‐length transcripts. In the presence of a high‐quality reference genome, genome‐guided approaches are preferred, as the reads are aligned directly to the target genome in advance. Aligned RNA evidence provides resolution on exon boundaries, and aids in the identification of splice variants. De novo approaches build graph models directly from the short (or long) reads to generate transcripts. The latter is much more challenging, computationally intensive, and prone to error.

We compared the accuracy of annotations produced by StringTie2, the accuracy of de novo–assembled transcripts with Trinity, and the accuracy of annotations produced by BRAKER. The selected packages are top performers when compared in their respective categories of genome‐guided and de novo transcriptome assembly (Sahraeian et al., 2017; Venturini et al., 2018). As expected, Trinity produced a higher number of transcripts than StringTie2, and BUSCO completeness was consistently lower (Table 3), except for *Liriodendron*. The gene models generated by StringTie2 were more numerous than the BRAKER gene models, resulting in more than was expected for each species. It should be noted, however, that StringTie2 identifies splice variants by generating a splice graph and resolving conflict between multiple potential splice sites (Kovaka et al., 2019), whereas BRAKER trains an internal algorithm, GeneMark‐ET, to find specific genes with complete support among all introns to be further used in training AUGUSTUS (Hoff et al., 2019).

The StringTie2 runs resulted in lower BUSCO completeness than the BRAKER and/or TSEBRA runs (Figure 4A, Appendix S12). This outcome is supported by the lack of ab initio prediction with genome‐guided approaches. Inflated mono‐exonic predictions (and lower BUSCO scores) were also observed in the StringTie2 genome annotation of the water strider (*Microvelia longipes*) (Toubiana et al., 2021). In our study, the *Rosa* ST2 (SR/LR) run was closest to the BRAKER runs, with a BUSCO score of 97.2%, BR (SR/LR) of 96.9%, and TSB (SR/ST2) of 98% (Appendices S10, S12).

### Genome annotations are improved when combining published full‐length protein sequences and read data

The performance of StringTie2 and the Trinity‐derived protein evidence was assessed on the predicted gene models using BRAKER and TSEBRA. In this context, the genome‐guided or de novo–assembled transcripts were translated into proteins and provided as evidence to train the ab initio component of the pipelines. Adding protein evidence to genome annotations can target protein‐coding genes, leading to more accurate predictions than RNA‐Seq evidence alone (Bruna, 2022). This study specifically focused on using protein evidence derived in some fashion from the transcriptomic inputs, but also evaluated the recommendation to include clade‐specific OrthoDB protein inputs to the BRAKER/TSEBRA approach.

The TSEBRA runs of the model species *Arabidopsis* and *Populus* were compared with the reference annotations. These runs were the best for the model species in terms of sensitivity and specificity as compared to the MAKER, StringTie2, Trinity, and BRAKER runs (Figure 4B). The model genomes also had very similar BUSCO completeness scores, but different mono:multi ratios with the addition of protein evidence. As expected, the model genomes benefited the most from the inclusion of the external OrthoDB proteins in terms of annotation rate and BUSCO score (both *Arabidopsis* and *Populus* reference proteins are contained within this resource); however, mono:multi ratio challenges remained consistent across the TSEBRA runs with varying inputs.

In the case of non‐model plant genomes, TSEBRA contributed to higher mono:multi ratios, which was very evident in *Liriodendron*; however, the BUSCO scores of the non‐protein runs were lower across all runs of *Liriodendron* than for all runs using protein evidence. The *Rosa* TSB (SR/OrthoDB) had the highest BUSCO score across all runs, which we believe may have been greatly influenced by the addition of OrthoDB, given the phylogenetic placement of *Rosa* in comparison to *Arabidopsis*. On the other hand, the annotation rate of TSB (SR/OrthoDB) was similar to that of the other runs in *Rosa*. The higher quality of the *Rosa* genome assembly, compared with the other two non‐models, could also influence the utility of the protein evidence; however, the mono:multi ratios remained high, and annotation rates were similar to those of the runs without OrthoDB proteins.

TSEBRA runs with proteins sourced from genome‐guided predictions performed similarly, but had lower BUSCO scores, higher mono:multi ratios, and lower total gene numbers when compared with the short read–only runs (Figure 4A, Appendix S10). Among the TSEBRA runs, Trinity fares better only for *Liriodendron*, which could indicate that genome‐guided proteins are not a suitable choice for a more repetitive genome. This is consistent with the independent assessments between the de novo transcriptome assemblers and genome‐guided assemblers with complex genomes with fragmented genome assemblies (e.g., in *Aedes albopictus* [Huang et al., 2016]). TSEBRA with full‐length proteins sourced from OrthoDB had lower BUSCO scores when compared to BR (SR) for the non‐model species *Liriodendron* and *Funaria*.

The total number of genes predicted by the TSEBRA and BRAKER runs remained largely the same across all species (Appendix S10). However, it should be noted that the number of mono‐exonic genes increased, whereas the multi‐exonic genes decreased, across all TSEBRA runs relative to the BRAKER runs without proteins across all species. The average gene lengths also decreased, while the average lengths of mono‐exonics remained the same, and the lengths of multi‐exonics were higher.

Our initial examination of the EnTAP reciprocal BLAST assessment revealed high annotation rates for the non‐model species when protein evidence was included, particularly the multi‐exonics (whereas the mono‐exonic percentage remained the same) (Appendix S10). However, this increase in multi‐exonic annotation proved to be an artifact resulting from the reduced numbers of these genes using this approach. Direct comparisons of the predictions revealed that 40–52% of the multi‐exonics were split into mono‐exonic predictions when comparing the BR (SR) to the TSB (SR/ST2) and TSB (SR/OrthoDB) gene models predicted using *Liriodendron* (Appendices S13, S14).

### Long reads can be paired with short reads to improve model quality

Long reads generated from platforms such as ONT or PacBio have the potential to resolve splice variants and assemble transcripts more accurately than traditional Illumina RNA‐Seq (Amarasinghe et al., 2020). While long reads can independently generate transcriptomes, it is recommended to have a combination of short and long reads to achieve greater depth, improved error profiles, and more evidence for splice site resolution (Gonzalez‐Ibeas et al., 2016; Watson and Warr, 2019; Amarasinghe et al., 2020).

In this study, we utilized both ONT PromethION and PacBio Iso‐Seq long reads. In the latter, we relied on raw reads (not the error‐corrected circular consensus sequencing reads) in our comparisons for genome annotations. In all cases, long reads (alone) did not outperform short reads for the BRAKER runs; however, in some cases, the combination of short‐read and long‐read inputs was beneficial. The Iso‐Seq reads from *Populus* and *Liriodendron* had a higher error rate and produced comparable, but lower, BUSCO scores than the BR (SR) runs. By contrast, the ONT PromethION long reads used for *Arabidopsis* and *Rosa* in the combined runs (BR (SR/LR)) had slightly better BUSCO completeness than the BR (SR) runs, and similar mono:multi ratios. Overall, the lower error profile of using ONT PromethION reads, supplemented with short‐read data, as well as the use of high‐quality reference genomes, support the higher BUSCO completeness scores.

### Best practices for plant genome annotation

From the existing tools, we recommend that investigators utilize RepeatModeler2 to mask their genome of interest with the default settings (Flynn et al., 2020). Following soft masking, RNA‐Seq short reads (between 4–10 libraries, minimum 15 million paired‐end reads per library) are generally sufficient for annotation. While we did not comprehensively investigate the impact of tissue type, it is recommended to sample from multiple tissues when possible (Kress et al., 2022). In our study, we did not observe a difference in the annotation completeness among species with a higher number of short‐read libraries, although we did not comprehensively evaluate the effect of using fewer libraries within a single species.

Sequencing long‐read libraries remains more expensive than generating deep Illumina short‐read RNA‐Seq coverage. In most cases, the short reads were a sufficient input. The notable exceptions include the BR (SR/LR), as they were comparable, and in some cases slightly better than, the BR (SR) runs across all species. The PromethION reads had a lower error profile and were more beneficial when combined with short reads; however, current long‐read technologies available from both platforms may provide different results.

BRAKER and TSEBRA outperformed runs of MAKER, StringTie2, and Trinity with default settings. It should be noted that we did not comprehensively benchmark MAKER with more than two training runs of AUGUSTUS (Appendix 15), as is recommended, which could have further improved results; however, previous benchmarking studies also reported a lower performance for MAKER (Banerjee et al., 2021; Brůna et al., 2021). Among the BRAKER runs executed in the model plants *Arabidopsis* and *Populus*, the TSEBRA runs were best. TSEBRA, especially TSB (SR/OrthoDB), performed best for *Rosa* but would require substantial filtering to remove false positives. Among the less contiguous and more evolutionary distant species (*Funaria* and *Liriodendron*), the BR (SR) runs performed best in terms of BUSCO completeness and mono:multi ratios. The overall EnTAP annotation rates were greatly improved in runs where OrthoDB proteins were included as evidence; however, when considering BUSCO scores and mono:multi ratios, especially for non‐model species, the BR (SR) runs performed best. For more divergent species (as defined by current public databases), BRAKER runs with short reads, or short reads and long reads, are advised.

Regardless of approach, the existing pipelines do not provide appropriate summary statistics or robust methods for filtering unlikely gene models. All methods produce more putative false positives than desired. We recommend utilizing reciprocal BLAST searches with well‐curated databases containing targets with full‐length proteins (such as NCBI's RefSeq) to identify fragmented models. We also recommend filtering and removing mono‐exonics that do not have a protein domain. Finally, we recommend structural filters to remove unlikely gene structures (e.g., splice sites, start sites, incompletes).

In this study, we demonstrated the impact of post‐filtering on the most complex genome assessed in this study, *Liriodendron*. We improved the published annotation across all benchmarks evaluated in this study following a new BR (SR) run (Table 4) (Chen et al., 2019). The filters reduced the overall number of putative false positives and increased the overall rate of annotation, with minimal reduction to BUSCO completeness.

## AUTHOR CONTRIBUTIONS

The genome annotation software was executed for benchmarking purposes with contributions from V.S.V., D.M., J.B., and B.S. Troubleshooting and statistical evaluation of the genome annotations were undertaken by C.W., A.B., K.C.F., S.Z., S.L.M., M.C., and J.L.W. J.L.W. was responsible for acquiring the funding. Data analysis and manuscript preparation were carried out by V.S.V., with assistance from C.W., K.C.F., S.Z., and J.L.W. All authors approved the final version of the manuscript.

### Open Research Badges

This article has earned Open Data and Open Materials badges. Data and materials are available at https://dx.doi.org/10.17504/protocols.io.yxmvmkp95g3p/v1.

## Supporting information


**Appendix S1.** Genomes for this study, with versions and links to the genome.

**Appendix S2.** Public transcriptomic evidence (short and long read).

**Appendix S3.** StringTie proteins before and after frame‐selection.

**Appendix S4.** Mono:multi ratios in published model plant genomes.

**Appendix S5.** Initial genome statistics.

**Appendix S6.** RNA alignments for short and long reads.

**Appendix S7.** N50s for the short and long reads.

**Appendix S8.** Sensitivity and precision scores for *Arabidopsis* and *Populus*.

**Appendix S9.** Gene characteristics in BRAKER runs.

**Appendix S10.** Comparisons among StringTie2, BRAKER, and TSEBRA runs.

**Appendix S11.** *Liriodendron* masked and unmasked.

**Appendix S12.** Comparison between StringTie2 and BRAKER runs.

**Appendix S13.** Overlaps between BR (SR) and BR (SR/ST2) in *Liriodendron*.

**Appendix S14.** Genes predicted as mono‐exonic from the BR (SR/ST2) run overlapping with multis from the BR (SR) run in *Liriodendron*.

**Appendix S15.** Comparison between MAKER and BRAKER.

## Data Availability

All scripts and data used are available through https://dx.doi.org/10.17504/protocols.io.yxmvmkp95g3p/v1. The public data (NCBI SRA and genome assembly accessions) for the reference genomes, short reads, and long reads are listed in Appendix S2.
