# Correction to Welcome to the big leaves: Best practices for improving genome annotation in non‐model plant genomes

**DOI:** 10.1002/aps3.11553

**Published:** 2023-11-07

Vuruputoor, V. S., D. Monyak, K. C. Fetter, C. Webster, A. Bhattarai, B. Shrestha, S. Zaman, et al. 2023. Welcome to the big leaves: Best practices for improving genome annotation in non‐model plant genomes. *Applications in Plant Sciences* 11(4): e11533.

Figure 4 in the published manuscript contained the following errors. Figure 4A and 4B were missing violin plots for MAKER, which should have been colored green. Figure 4C incorrectly displayed the ideal range of scores—the yellow bar should have spanned 85 to 100 instead of the range shown. Additionally, the color scheme was incorrect. The BRAKER runs should have been colored blue and the StringTie2 runs should have been red. The corrected Figure 4 is presented here with its original caption, which was correct.

**Figure 4:**
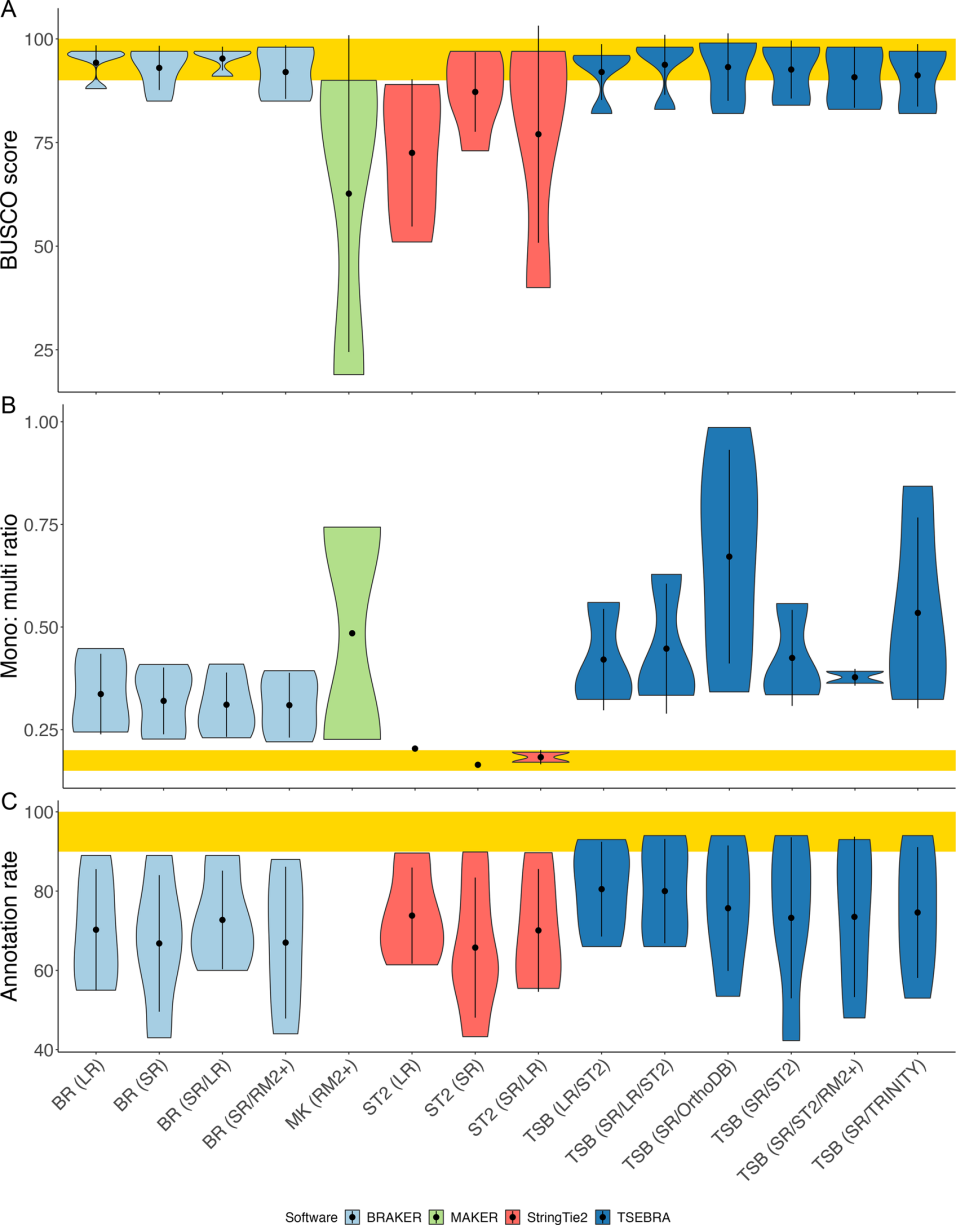
Comparison of scores across all species between the runs of different input types and software. (A) BUSCO completeness scores. (B) Mono: multi ratios. (C) EnTAP annotation rates. MAKER is shown in green, BRAKER is light blue, TSEBRA is dark blue, and StringTie2 is red. The yellow rectangle represents the target scores for each benchmark. RM2+, RepeatModeler2 with LTRStruct.

We apologize for this error.

