# Supplementary material for: Welcome to the big leaves: Best practices for improving genome annotation in non‐model plant genomes
Source: Appl Plant Sci. 2023 Aug 8;11(4):e11533. doi: 10.1002/aps3.11533 (PMC10439824; doi:10.1002/aps3.11533)
Supplement: Supplementary file 1 — Appendix S1. Genomes for this study, with versions and links to the genome. [file APS3-11-e11533-s002.docx]

**Appendix S1.** Genomes for this study, with versions and links to the genome.

| **Species** | **Paper** | **Version** | **Link to Genome** |
| --- | --- | --- | --- |
| *Arabidopsis thaliana* | (Lamesch et al. 2012) | TAIR10 - release 41 | ftp://ftp.ensemblgenomes.org/pub/plants/release-41/fasta/arabidopsis_thaliana/dna/Arabidopsis_thaliana.TAIR10.dna_sm.toplevel.fa.gz |
| *Funaria hygrometrica* | (Kirbis et al. 2022) | Version 1 | Inhouse |
| *Populus trichocarpa* | (Tuskan et al. 2006) | Version 3 | ftp://ftp.ensemblgenomes.org/pub/plants/release-49/fasta/populus_trichocarpa/dna/ |
| *Liriodendron chinense* | (Chen et al. 2019) | Version 1 | https://hardwoodgenomics.org/sites/default/files/sequences/liriodendron_chinense/LIR.pbjelly.reN.final.fasta |
| *Rosa chinensis* | (Raymond et al. 2018) | Version 1 | https://www.rosaceae.org/rosaceae_downloads/Rosa_chinensis/Rchinensis_Old_Blush_homozygous_genome-v2.0/assembly/Rosa_chinensis_Old_Blush_homozygous_genome-v2.0.fna.gz |

**REFERENCES**

Chen, J., Z. Hao, X. Guang, C. Zhao, P. Wang, L. Xue, Q. Zhu, et al. 2019. *Liriodendron* genome sheds light on angiosperm phylogeny and species-pair differentiation. *Nature Plants* 5(1): 18–25.

Kirbis, A., N. Rahmatpour, S. Dong, J. Yu, N. van Gessel, M. Waller, R. Reski, et al. 2022. Genome dynamics in mosses: Extensive synteny coexists with a highly dynamic gene space. bioRxiv 492078 [Preprint] [posted 18 May 2022]. Available at <https://doi.org/10.1101/2022.05.17.492078> [accessed 12 May 2023].

Lamesch, P., T. Z. Berardini, D. Li, D. Swarbreck, C. Wilks, R. Sasidharan, R. Muller, et al. 2012. The Arabidopsis Information Resource (TAIR): Improved gene annotation and new tools. *Nucleic Acids Research* 40(Database issue): D1202–D1210.

Raymond, O., J. Gouzy, J. Just, H. Badouin, M. Verdenaud, A. Lemainque, P. Vergne, et al. 2018. The *Rosa* genome provides new insights into the domestication of modern roses. *Nature Genetics* 50(6): 772–777.

Tuskan, G. A., S. Difazio, S. Jansson, J. Bohlmann, I. Grigoriev, U. Hellsten, N. Putnam, et al. 2006. The genome of black cottonwood, *Populus trichocarpa* (Torr. & Gray). *Science* 313(5793): 1596–1604.
