# Supplementary material for: Welcome to the big leaves: Best practices for improving genome annotation in non‐model plant genomes
Source: Appl Plant Sci. 2023 Aug 8;11(4):e11533. doi: 10.1002/aps3.11533 (PMC10439824; doi:10.1002/aps3.11533)
Supplement: Supplementary file 2 — Appendix S2. Public transcriptomic evidence (short and long read). [file APS3-11-e11533-s013.docx]

**Appendix S2.** Public transcriptomic evidence (short and long read).

| **Species** | **BioProject #** | **SRA (SRP/ERP) Study** | **SRX/ERX Experiment** | **SRR/ERR Run** | **Spots (# read locations)** | **Bases** | **Bytes** | **Tissue type** | **Sequencer** | **Paired/single** |
| --- | --- | --- | --- | --- | --- | --- | --- | --- | --- | --- |
| ***Funaria hygrometrica*** | PRJNA421369 | SRP126294 | SRX3452863 | SRR8527271 | 49,324,732 | 7.4G | 2.5Gb | Haploid gametophyte | Illumina NextSeq 500 | paired |
|  |  |  | SRX5330016 | SRR6356272 | 34,002,818 | 5.1G | 1.7Gb | Haploid gametophyte | Illumina NextSeq 500 | paired |
|  |  |  | SRX5330017 | SRR8527272 | 37,100,201 | 5.6G | 1.9Gb | Haploid gametophyte | Illumina NextSeq 500 | paired |
| ***Arabidopsis thaliana*** | PRJNA284739 | SRP058628 | SRX1036705 | SRR2037363 | 82,831,218 | 16.6G | 10.6Gb | Stem | Illumina HiSeq 2000 | paired |
|  | PRJNA284739 | SRP058628 | SRX1036705 | SRR2037362 | 39,299,841 | 7.9G | 5.1Gb | Stem | Illumina HiSeq 2000 | paired |
|  | PRJNA284739 | SRP058628 | SRX1036693 | SRR2037338 | 57,633,045 | 11.5G | 7.5Gb | Stem | Illumina HiSeq 2000 | paired |
|  | PRJNA284739 | SRP058628 | SRX1036695 | SRR2037346 | 64,896,842 | 13G | 8.5Gb | Leaf | Illumina HiSeq 2000 | paired |
|  | PRJEB14117 | ERP015730 | ERX2770903 | ERR2757910 | 36,319,987 | 10.3G | 4Gb | Leaf | Illumina HiSeq 2000 | paired |
|  | PRJNA340889 | SRP083317 | SRX2065453 | SRR4095612 | 17,676,726 | 3.6G | 1.5Gb | Seeds | Illumina HiSeq 2500 | paired |
|  | PRJNA340889 | SRP083317 | SRX2065448 | SRR4095607 | 14,959,896 | 3G | 1.3Gb | Seeds | Illumina HiSeq 2500 | paired |
|  | PRJEB42556 | ERP126434 | ERX5132034 | ERR5343646 | 10,151,920 | 3G | 1.2Gb | Aerial Part | Illumina HiSeq 3000 | paired |
|  | PRJEB40453 | ERP124098 | ERX4548377 | ERR4619338 | 24,106,303 | 7.3G | 3Gb | 17 day-old shoots | Illumina HiSeq 3000 | paired |
|  | PRJNA594286 | SRP235227 | SRX7290465 | SRR10611193 | 8,258,511 | 10.1G | 8Gb | Unkown | Oxford Nanopore PromethION | single |
|  | PRJNA594286 | SRP235227 | SRX7290466 | SRR10611194 | 7,849,574 | 9.8G | 7.8Gb | Unkown | Oxford Nanopore PromethION | single |
|  | PRJNA594286 | SRP235227 | SRX7290467 | SRR10611195 | 7,025,983 | 8.6G | 6.8Gb | Unkown | Oxford Nanopore PromethION | single |
|  | PRJNA725066 | SRP316288 | SRX10677446 | SRR14322310 | 68,785,614 | 20.6G | 6.1Gb | axillary buds | Illumina NovaSeq 6000 | paired |
| ***Populus trichocarpa*** | PRJNA515420 | SRP179723 | SRX9893893 | SRR13481183 | 21,263,642 | 5.3G | 1.8Gb | Secondary xylem | Illumina HiSeq 2500 | paired |
|  | PRJNA628501 | SRP258577 | SRX8177635 | SRR11611372 | 13,991,070 | 4.2G | 1.4Gb | seedling needles | Illumina HiSeq 2500 | paired |
|  | PRJNA650996 | SRP289313 | SRX9383942 | SRR12919313 | 22,476,947 | 6.8G | 2Gb | bark | Illumina NovaSeq 6000 | paired |
|  | PRJNA696790 | SRP306056 | SRX10084749 | SRR13695406 | 20,039,851 | 6.1G | 1.8Gb | leaf | Illumina NovaSeq 6000 | paired |
|  | PRJNA725066 | SRP316288 | SRX10677446 | SRR14322310 | 68,785,614 | 20.6G | 6.1Gb | axillary buds | Illumina NovaSeq 6000 | paired |
|  | PRJNA516416 | SRP179723 | SRX5254305 | SRR8447264 | 161,334 | 383.7M | 93.1Mb | secondary xylem | PacBio SMRT | single |
| ***Liriodendron chinense*** | PRJNA559687 | SRP218024 | SRX6693922 | SRR9945429 | 45,919,256 | 13.8G | 4Gb | bract | Illumina HiSeq 2500 | paired |
|  |  |  | SRX6697492 | SRR9949010 | 38,265,327 | 11.5G | 3.3Gb | bract | Illumina HiSeq 2500 | paired |
|  |  |  | SRX6693921 | SRR9945430 | 39,896,641 | 12G | 3.4Gb | leaf | Illumina HiSeq 2500 | paired |
|  |  |  | SRX6697491 | SRR9949011 | 34,672,556 | 10.4G | 2.9Gb | leaf | Illumina HiSeq 2500 | paired |
|  |  |  | SRX6697402 | SRR9948916 | 41,436,299 | 12.4G | 3.6Gb | leaf | Illumina HiSeq 2500 | paired |
|  |  |  | SRX6693920 | SRR9945431 | 38,819,287 | 11.6G | 3.4Gb | petal | Illumina HiSeq 2500 | paired |
|  |  |  | SRX6697494 | SRR9949008 | 29,694,072 | 8G | 2.3Gb | petal | Illumina HiSeq 2500 | paired |
|  |  |  | SRX6697399 | SRR9948919 | 37,405,592 | 11.2G | 3.2Gb | petal | Illumina HiSeq 2500 | paired |
|  |  |  | SRX6693919 | SRR9945432 | 30,029,905 | 9G | 2.6Gb | pistil | Illumina HiSeq 2500 | paired |
|  |  |  | SRX6697493 | SRR9949009 | 41,125,315 | 12.3G | 3.5Gb | pistil | Illumina HiSeq 2500 | paired |
|  |  |  | SRX6697400 | SRR9948918 | 34,728,258 | 10.4G | 3Gb | pistil | Illumina HiSeq 2500 | paired |
|  |  |  | SRX6693918 | SRR9945433 | 44,447,597 | 13.3G | 3.8Gb | shoot apex | Illumina HiSeq 2500 | paired |
|  |  |  | SRX6697496 | SRR9949006 | 37,881,676 | 11.4G | 3.3Gb | shoot apex | Illumina HiSeq 2500 | paired |
|  |  |  | SRX6697404 | SRR9948914 | 37,425,347 | 11.2G | 3.2Gb | shoot apex | Illumina HiSeq 2500 | paired |
|  |  |  | SRX6693917 | SRR9945434 | 45,653,579 | 13.7G | 3.9Gb | sepal | Illumina HiSeq 2500 | paired |
|  |  |  | SRX6697495 | SRR9949007 | 43,209,095 | 13G | 3.7Gb | sepal | Illumina HiSeq 2500 | paired |
|  |  |  | SRX6697405 | SRR9948913 | 33,151,069 | 9.9G | 2.8Gb | sepal | Illumina HiSeq 2500 | paired |
|  |  |  | SRX6693916 | SRR9945435 | 46,620,902 | 14G | 4Gb | stamen | Illumina HiSeq 2500 | paired |
|  |  |  | SRX6697497 | SRR9949005 | 38,971,271 | 11.7G | 3.3Gb | stamen | Illumina HiSeq 2500 | paired |
|  |  |  | SRX6697403 | SRR9948915 | 41,065,829 | 12.3G | 3.5Gb | stamen | Illumina HiSeq 2500 | paired |
|  | PRJNA665077 | SRP285036 | SRX9174881 | SRR12695187 | 10,437,029 | 22.7G | 5.4Gb | All 7 samples above pooled together | PACBIO-SMRT | single |
| ***Rosa chinensis*** | PRJNA398090 | SRP115334 | SRX6490239 | SRR9733228 | 37,170,059 | 11.2G | 3.2Gb | the abaxial side of petal | Illumina HiSeq 4000 | paired |
|  | PRJNA473465 | SRP150297 | SRX4195946 | SRR7293274 | 35,120,535 | 10.5G | 3.9Gb | leaves | Illumina HiSeq 2000 | paired |
|  | PRJNA473465 | SRP150297 | SRX4195940 | SRR7293280 | 33,483,963 | 10G | 3.8Gb | leaves | Illumina HiSeq 2000 | paired |
|  | PRJNA546486 | SRP200448 | SRX5973706 | SRR9202384 | 30,805,477 | 9.2G | 2.7Gb | Stamen | Illumina HiSeq 4000 | paired |
|  | PRJNA546486 | SRP200448 | SRX5973701 | SRR9202389 | 29,793,315 | 8.9G | 2.7Gb | Pistil_Ovary | Illumina HiSeq 4000 | paired |
|  | PRJNA546486 | SRP200448 | SRX5973705 | SRR9202385 | 28,535,324 | 8.6G | 2.5Gb | Prickle | Illumina HiSeq 4000 | paired |
|  | PRJNA596583 | SRP238318 | SRX7419418 | SRR10744004 | 22,985,180 | 6.8G | 2Gb | leaf | Illumina HiSeq 4000 | paired |
|  | PRJNA414720 | SRP120300 | SRX3296708 | SRR6186662 | 23,141,140 | 6.7G | 2.3Gb | leaf | Illumina HiSeq 2500 | paired |
|  | PRJNA414720 | SRP120300 | SRX3296710 | SRR6186660 | 27,768,588 | 5G | 1.9Gb | shoot apical meristem | Illumina HiSeq 2500 | paired |
|  | PRJNA236618 | SRP035933 | SRX451091 | SRR1145848 | 33,261,614 | 4.8G | 2.6Gb | Flower bud, open flower and senescent flower | Illumina HiSeq 2000 | paired |
|  |  |  | SRX9153921 | SRR12673743 | 7,290,141 | 8.2G | 6.9Gb |  |  |  |
|  |  |  | SRX9153918 | SRR12673746 | 6,924,721 | 8.7G | 7.3Gb |  |  |  |
|  |  |  | SRX9153916 | SRR12673748 | 6,747,461 | 7G | 5.9Gb |  |  |  |
|  |  |  | SRX9153914 | SRR12673750 | 6,585,762 | 7.3G | 6.1Gb |  |  |  |
|  |  |  | SRX9153912 | SRR12673752 | 7,641,480 | 9.1G | 7.6Gb |  |  |  |
|  |  |  | SRX9153909 | SRR12673755 | 6,739,818 | 8G | 6.7Gb |  |  |  |
