# Supplementary material for: Welcome to the big leaves: Best practices for improving genome annotation in non‐model plant genomes
Source: Appl Plant Sci. 2023 Aug 8;11(4):e11533. doi: 10.1002/aps3.11533 (PMC10439824; doi:10.1002/aps3.11533)
Supplement: Supplementary file 3 — Appendix S3. StringTie proteins before and after frame‐selection. [file APS3-11-e11533-s005.docx]

**Appendix S3.** StringTie proteins before and after frame-selection.

|  |  | **BUSCO on transdecoder protein** | **BUSCO on gFACs protein** |
| --- | --- | --- | --- |
| ***Arabidopsis*** | SR | C:93.9%[S:67.4%,D:26.5%],F:1.4%,M:4.7%,n:1614 | C:39.8%[S:29.7%,D:10.1%],F:7.5%,M:52.7%,n:1614 |
|  | LR | C:85.5%[S:61.3%,D:24.2%],F:5.0%,M:9.5%,n:1614 | C:34.9%[S:27.1%,D:7.8%],F:4.8%,M:60.3%,n:1614 |
|  | SR+LR | C:94.5%[S:65.1%,D:29.4%],F:0.4%,M:5.1%,n:1614 | C:40.9%[S:31.2%,D:9.7%],F:4.8%,M:54.3%,n:1614 |
|  | SR (RM2+) | C:96.4%[S:70.9%,D:25.5%],F:0.8%,M:2.8%,n:1614 | C:41.5%[S:31.9%,D:9.6%],F:5.5%,M:53.0%,n:1614 |
| ***Funaria*** | SR | C:86.7%[S:41.6%,D:45.1%],F:2.4%,M:10.9%,n:1614 | C:47.1%[S:30.4%,D:16.7%],F:5.9%,M:47.0%,n:1614 |
|  | SR (RM2+) | C:86.7%[S:41.6%,D:45.1%],F:2.4%,M:10.9%,n:1614 | C:47.1%[S:30.4%,D:16.7%],F:5.9%,M:47.0%,n:1614 |
| ***Populus*** | SR | C:95.8%[S:57.2%,D:38.6%],F:1.1%,M:3.1%,n:1614 | C:42.8%[S:30.2%,D:12.6%],F:9.8%,M:47.4%,n:1614 |
|  | LR | C:52.3%[S:42.4%,D:9.9%],F:13.0%,M:34.7%,n:1614 | C:22.4%[S:19.6%,D:2.8%],F:7.0%,M:70.6%,n:1614 |
|  | SR +LR | C:86.1%[S:65.3%,D:20.8%],F:7.8%,M:6.1%,n:1614 | C:35.0%[S:30.2%,D:4.8%],F:6.7%,M:58.3%,n:1614 |
|  | SR (RM2+) | C:95.8%[S:57.2%,D:38.6%],F:1.1%,M:3.1%,n:1614 | C:42.8%[S:30.2%,D:12.6%],F:9.8%,M:47.4%,n:1614 |
| ***Liriodendron*** | SR | C:89.2%[S:52.7%,D:36.5%],F:6.4%,M:4.4%,n:1614 |  |
|  | LR | C:66.8%[S:53.0%,D:13.8%],F:13.2%,M:20.0%,n:1614 | C:26.4%[S:21.7%,D:4.7%],F:6.6%,M:67.0%,n:1614 |
|  | SR +LR | C:79.1%[S:59.5%,D:19.6%],F:12.9%,M:8.0%,n:1614 | C:35.3%[S:29.9%,D:5.4%],F:9.0%,M:55.7%,n:1614 |
|  | SR (RM2+) | C:89.2%[S:52.7%,D:36.5%],F:6.4%,M:4.4%,n:1614 | C:43.6%[S:29.7%,D:13.9%],F:11.7%,M:44.7%,n:1614 |
| ***Rosa*** | SR | C:97.7%[S:68.0%,D:29.7%],F:0.6%,M:1.7%,n:1614 | C:43.7%[S:33.0%,D:10.7%],F:7.6%,M:48.7%,n:1614 |
|  | LR | C:89.7%[S:57.2%,D:32.5%],F:4.3%,M:6.0%,n:1614 | C:36.6%[S:27.4%,D:9.2%],F:7.3%,M:56.1%,n:1614 |
|  | SR +LR | C:98.0%[S:49.6%,D:48.4%],F:0.4%,M:1.6%,n:1614 | C:47.2%[S:31.8%,D:15.4%],F:9.5%,M:43.3%,n:1614 |
