# Supplementary material for: Welcome to the big leaves: Best practices for improving genome annotation in non‐model plant genomes
Source: Appl Plant Sci. 2023 Aug 8;11(4):e11533. doi: 10.1002/aps3.11533 (PMC10439824; doi:10.1002/aps3.11533)
Supplement: Supplementary file 4 — Appendix S4. Mono:multi ratios in published model plant genomes. [file APS3-11-e11533-s006.docx]

**Appendix S4.** Mono:multi ratios in published model plant genomes.

| **Species** | **Mono** | **Multi** | **Total** | **Ratio** | **Source** |
| --- | --- | --- | --- | --- | --- |
| **Arabidopsis** | 8093 | 40227 | 48320 | 0.20 | ftp://ftp.ensemblgenomes.org/pub/plants/release-41/fasta/arabidopsis_thaliana/dna/Arabidopsis_thaliana.TAIR10.dna_sm.toplevel.fa.gz |
| **Populus** | 10802 | 62210 | 73012 | 0.17 | ftp://ftp.ensemblgenomes.org/pub/plants/release-49/fasta/populus_trichocarpa/dna/ |
| ***Oryza sativa*** | 8825 | 33715 | 42540 | 0.26 | <https://ftp.ncbi.nlm.nih.gov/genomes/all/GCF/001/433/935/GCF_001433935.1_IRGSP-1.0/GCF_001433935.1_IRGSP-1.0_genomic.fna.gz> |
| ***Solanum lycopersicum*** | 6123 | 31460 | 37583 | 0.19 | <https://ftp.ncbi.nlm.nih.gov/genomes/all/GCF/000/188/115/GCF_000188115.5_SL3.1/GCF_000188115.5_SL3.1_genomic.fna.gz> |
| ***Nicotiana tabacum*** | 15220 | 69003 | 84223 | 0.22 | <https://ftp.ncbi.nlm.nih.gov/genomes/all/GCF/000/715/135/GCF_000715135.1_Ntab-TN90/GCF_000715135.1_Ntab-TN90_genomic.fna.gz> |
