# Supplementary material for: Welcome to the big leaves: Best practices for improving genome annotation in non‐model plant genomes
Source: Appl Plant Sci. 2023 Aug 8;11(4):e11533. doi: 10.1002/aps3.11533 (PMC10439824; doi:10.1002/aps3.11533)
Supplement: Supplementary file 5 — Appendix S5. Initial genome statistics. [file APS3-11-e11533-s014.docx]

**Appendix S5.** Initial genome statistics.

| **Species** | | ***Arabidopsis*** | ***Funaria*** | ***Liriodendron*** | ***Populus*** | ***Rosa*** |
| --- | --- | --- | --- | --- | --- | --- |
| **Genome size (bp)** | | 119,667,750 | 326,856,579 | 1,742,423,874 | 434,132,815 | 515,588,973 |
| **# of contigs** | | 7 | 687 | 3711 | 1446 | 55 |
| **N50** | | 23,459,830 | 1,484,274 | 3,525,943 | 19,465,461 | 69,643,165 |
| **Repeat content** | | 23.6 | 42.35 | 73.18 | 35.9 | 60.53 |
| **Repeat content (RM2+)** | | 16.51 | 43.12 | 72.66 | 45.06 |  |
| **BUSCO (genome)** | | C:99.3%[S:98.6%,D:0.7%],F:0.2%,M:0.5%,n:1614 | C:85.6%[S:73.5%,D:12.1%],F:2.4%,M:12.0%,n:1614 | C:98.6%[S:92.1%,D:6.5%],F:0.8%,M:0.6%,n:161 | C:98.8%[S:80.6%,D:18.2%],F:0.6%,M:0.6%,n:1614 | C:98.8%[S:94.4%,D:4.4%],F:0.6%,M:0.6%,n:1614 |
| **BUSCO**  **(annotated proteins)** | | C:99.6%[S:55.8%,D:43.8%],F:0.1%,M:0.3%,n:161 | C:86.6%[S:72.6%,D:14.0%],F:2.3%,M:11.1%,n:1614 | C:75.1%[S:68.3%,D:6.8%],F:15.1%,M:9.8%,n:1614 | C:98.3%[S:35.0%,D:63.3%],F:0.9%,M:0.8%,n:1614 | C:97.3%[S:93.4%,D:3.9%],F:1.7%,M:1.0%,n:1614 |
| **gFACs (reference annotation)** | **Mono** | 8093 | 15640 | 14521 | 10802 | 15383 |
|  | **Multi** | 40227 | 20620 | 20740 | 62210 | 35004 |
|  | **Total** | 48320 | 36260 | 35261 | 73012 | 50387 |
|  | **Ratio** | 0.20 | 0.76 | 0.70 | 0.17 | 0.44 |
