# Supplementary material for: Welcome to the big leaves: Best practices for improving genome annotation in non‐model plant genomes
Source: Appl Plant Sci. 2023 Aug 8;11(4):e11533. doi: 10.1002/aps3.11533 (PMC10439824; doi:10.1002/aps3.11533)
Supplement: Supplementary file 6 — Appendix S6. RNA alignments for short and long reads. [file APS3-11-e11533-s015.docx]

**Appendix S6.** RNA alignments for short and long reads.

| **Reference genome** | **RNA read alignments, reference species (%)** | | **De novo transcripts aligned to genome** | |
| --- | --- | --- | --- | --- |
|  | **Short-reads** | **Long-reads (minimap2-sequencer)** | **(% aligned) mono/multi** | **BUSCO Completeness** |
| *Funaria* | 97.13 | N/A | [94.3] 15179/39149 | C:57.2%[S:43.6%,D:13.6%],F:13.6%,M:29.2%,n:1614 |
| *Arabidopsis* | 97.06 | 97.1 (ONT) | [94.7] 11291/34492 | C:78.8%[S:67.5%,D:11.3%],F:13.6%,M:7.6%,n:1614 |
| *Populus* | 91.41 | 92.01 (PacBio) | [73.7] 21604/57170 | C:78.7%[S:52.0%,D:26.7%],F:12.7%,M:8.6%,n:1614 |
| *Liriodendron* | 97.05 | 95.5 (PacBio) | [69.6] 66629/79698 | C:76.5%[S:47.1%,D:29.4%],F:13.8%,M:9.7%,n:1614 |
| *Rosa* | 91.96 | 99 (ONT) | [72.8] 29985/65031 | C:83.2%[S:57.4%,D:25.8%],F:12.0%,M:4.8%,n:1614 |
