# Supplementary material for: Welcome to the big leaves: Best practices for improving genome annotation in non‐model plant genomes
Source: Appl Plant Sci. 2023 Aug 8;11(4):e11533. doi: 10.1002/aps3.11533 (PMC10439824; doi:10.1002/aps3.11533)
Supplement: Supplementary file 7 — Appendix S7. N50s for the short and long reads. [file APS3-11-e11533-s004.docx]

**Appendix S7.** N50s for the short and long reads.

| **Species** | **SR- Total reads (N50)** | **LR- Total reads (N50)** |
| --- | --- | --- |
| ***Arabidopsis*** | 52,912,443,562 (100) | 72,435,151,489 (2249) |
| ***Liriodendron*** | 208,110,232,535 (150) | 22,713,962,011 (4673) |
| ***Populus*** | 53,390,254,188 (150) | 558,593,446 (1,348) |
| ***Rosa*** | 135,933,621,273 (150) | 482,704,44,525 (976) |
| ***Funaria*** | 43,506,430,892 (76) |  |
