# Supplementary material for: Welcome to the big leaves: Best practices for improving genome annotation in non‐model plant genomes
Source: Appl Plant Sci. 2023 Aug 8;11(4):e11533. doi: 10.1002/aps3.11533 (PMC10439824; doi:10.1002/aps3.11533)
Supplement: Supplementary file 8 — Appendix S8. Sensitivity and precision scores for Arabidopsis and Populus. [file APS3-11-e11533-s003.docx]

**Appendix S8.** Sensitivity and precision scores for *Arabidopsis* and *Populus.*

| **Species** | **Run** | **Sensitivity** | **Precision** |
| --- | --- | --- | --- |
| **Arabidopsis** | MK (RM2+) | 6.41 | 1.85 |
|  | BR (SR) | 40.37 | 60.11 |
|  | TSB (SR/TRINITY) | 55.94 | 80.78 |
|  | TSB (SR/ST2) | 56.94 | 81.92 |
|  | BR (SR/LR) | 42.45 | 61.88 |
|  | TSB (SR/LR/ST2) | 56.21 | 82.40 |
|  | BR (LR) | 42.46 | 59.79 |
|  | TSB (LR/ST2) | 54.91 | 81.17 |
|  | BR (SR/RM2+) | 43.67 | 61.70 |
|  | TSB (SR/RM2+/ST2) | 56.99 | 81.93 |
|  | ST2 (SR) | 50.07 | 63.24 |
|  | ST2 (LR/SR) | 46.41 | 51.99 |
|  | ST2 (LR) | 39.57 | 50.14 |
|  | TSB (SR/OrthoDB) | 58.5 | 80.85 |
| **Populus** | MK (RM2+) | 0.40 | 0.31 |
|  | BR (SR) | 32.01 | 41.02 |
|  | TSB (SR/TRINITY) | 39.50 | 62.58 |
|  | TSB (SR/ST2) | 41.83 | 62.77 |
|  | BR (SR/LR) | 31.87 | 40.79 |
|  | TSB (SR/LR/ST2) | 41.55 | 62.67 |
|  | BR (LR) | 32.45 | 42.23 |
|  | TSB (LR/ST2) | 39.85 | 62.82 |
|  | BR (SR/RM2+) | 31.73 | 41.25 |
|  | TSB (SR/RM2+/ST) | 41.55 | 60.56 |
|  | ST2 (SR) | 29.86 | 45.43 |
|  | ST2 (LR/SR) | 13.08 | 56.89 |
|  | ST2 (LR) | 19.96 | 56.68 |
|  | TSB (SR/OrthoDB) | 40.28 | 57.24 |
