# Supplementary material for: Welcome to the big leaves: Best practices for improving genome annotation in non‐model plant genomes
Source: Appl Plant Sci. 2023 Aug 8;11(4):e11533. doi: 10.1002/aps3.11533 (PMC10439824; doi:10.1002/aps3.11533)
Supplement: Supplementary file 9 — Appendix S9. Gene characteristics in BRAKER runs. [file APS3-11-e11533-s001.docx]

**Appendix S9.** Gene characteristics in BRAKER runs.

| **Species** | **Runs** |  | **Gene length** | |  |
| --- | --- | --- | --- | --- | --- |
|  |  | **Average gene length** | **mono** | **multi** | **Median number of exons per multiexonic gene:** |
| *Arabidopsis* | **BR (SR)** | 2936.07 | 854.67 | 3408.93 | 5 |
|  | **BR (LR)** | 2229.38 | 836.10 | 2569.73 | 5 |
|  | **BR (SR/LR)** | 2059.37 | 849.09 | 2338.11 | 5 |
|  | **TSB (SR/ST2)** | 1860.28 | 876.37 | 2245.72 | 5 |
|  | **TSB (SR/TRINITY)** | 1860.28 | 876.37 | 2245.72 | 5 |
|  | **TSB (LR/ST2)** | 1945.61 | 881.37 | 2348.70 | 5 |
|  | **TSB (SR/LR/ST2)** | 1957.18 | 885.25 | 2364.78 | 5 |
|  | **BR (SR/RM2+)** | 3629.76 | 892.27 | 4233.12 | 5 |
|  | **TSB (SR/ST2/RM2+)** | 1942.28 | 887.28 | 2355.87 | 5 |
|  | **TSB (SR/OrthoDB)** | 1929.71 | 877.52 | 2289.58 | 5 |
| *Funaria* | **BR (SR)** | 2112.89 | 592.61 | 2734.37 | 4 |
|  | **TSB (SR/ST2)** | 1701.69 | 627.82 | 3008.18 | 5 |
|  | **TSB (SR/TRINITY)** | 2061.61 | 586.63 | 3304.85 | 6 |
|  | **BR (SR/RM2+)** | 2124.45 | 604.88 | 2722.5 | 4 |
|  | **TSB (SR/ST2/RM2+)** | 1702.21 | 624.92 | 3007.26 | 5 |
|  | **TSB (SR/OrthoDB)** | 2072.513 | 584.954 | 3300.495 | 6 |
| *Liriodendron* | **BR (SR)** | 9399.97 | 682.3 | 12415.27 | 4 |
|  | **BR (LR)** | 8601.03 | 675.74 | 12149.00 | 4 |
|  | **BR (SR/LR)** | 9725.47 | 694.18 | 12734.64 | 4 |
|  | **TSB (SR/ST2)** | 6596.95 | 734.05 | 12274.35 | 5 |
|  | **TSB (SR/TRINITY)** | 6229.90 | 724.99 | 11872.86 | 4 |
|  | **TSB (LR/ST2)** | 6409.38 | 737.48 | 12204.38 | 4 |
|  | **BR (SR/RM2+)** | 9268.75 | 654.36 | 12326.227 | 4 |
|  | **TSB (SR/LR/ST2)** | 6238.68 | 733.26 | 11909.92 | 4 |
|  | **TSB (SR/RM2+/ST2)** | 6271.92 | 718.36 | 12040.91 | 4 |
|  | **TSB (SR/OrthoDB)** | 6886.243 | 732.22 | 12954.44 | 5 |
| *Populus* | **BR (SR)** | 3142.98 | 800.94 | 3709.66 | 4 |
|  | **BR (LR)** | 6963.77 | 792.77 | 8734.16 | 4 |
|  | **BR (SR/LR)** | 3970.86 | 748.12 | 4859.46 | 4 |
|  | **TSB (SR/ST2)** | 2724.53 | 841.97 | 3610.33 | 4 |
|  | **TSB (SR/TRINITY)** | 2764.77 | 831.73 | 3706.87 | 4 |
|  | **TSB (LR/ST2)** | 2703.65 | 818.37 | 3641.62 | 4 |
|  | **TSB (SR/LR/ST2)** | 2719.63 | 843.70 | 3599.58 | 4 |
|  | **BR (SR/RM2+)** | 4596.54 | 735.48 | 5678.38 | 4 |
|  | **BR (SR/ST2/RM2+)** | 2648.45 | 784.78 | 3593.29 | 4 |
|  | **TSB (SR/OrthoDB)** | 2864.61 | 796.04 | 3590.612 | 5 |
| *Rosa* | **BR (SR)** | 2372.42 | 723.95 | 2993.17 | 4 |
|  | **BR (LR)** | 3765.28 | 752.45 | 4952.92 | 4 |
|  | **BR (SR/LR)** | 3977.25 | 712.35 | 5289.26 | 4 |
|  | **TSB (SR/ST2)** | 2064.18 | 777.14 | 3033.22 | 4 |
|  | **TSB (SR/TRINITY)** | 2037.87 | 766.38 | 3010.40 | 4 |
|  | **TSB (LR/ST2)** | 3765.28 | 752.45 | 4952.92 | 4 |
|  | **TSB (SR/LR/ST2)** | 2059.58 | 791.41 | 3012.58 | 4 |
|  | **TSB (SR/OrthoDB)** | 2065.471 | 77.69 | 2997.96 | 5 |
