# Supplementary material for: Welcome to the big leaves: Best practices for improving genome annotation in non‐model plant genomes
Source: Appl Plant Sci. 2023 Aug 8;11(4):e11533. doi: 10.1002/aps3.11533 (PMC10439824; doi:10.1002/aps3.11533)
Supplement: Supplementary file 10 — Appendix S10. Comparisons among StringTie2, BRAKER, and TSEBRA runs. [file APS3-11-e11533-s012.docx]

**Appendix S10.** Comparisons among StringTie2, BRAKER, and TSEBRA runs.

| ***Species*** | **Runs** | **Total** | **Annotated genes (70/70)** | **Annotation rate** | **Percentage gene family assignment** | **mono** | **multi** | **ratio** | **monos annotated** | **multi annotated** | **%monos annotated** | **%multis annotated** | **busco** |
| --- | --- | --- | --- | --- | --- | --- | --- | --- | --- | --- | --- | --- | --- |
| ***Arabidopsis*** | BR (SR) | 27365 | 24594 | 89.87 | 0.96 | 5065 | 22296 | 0.23 | 4461 | 20129 | 88.07 | 90.28 | C:96.3%[S:90.2%,D:6.1%],F:1.0%,M:2.7%,n:1614 |
|  | BR (LR) | 28469 | 25512 | 89.61 | 0.96 | 5589 | 22880 | 0.24 | 4812 | 20700 | 86.10 | 90.47 | C:97.1%[S:87.3%,D:9.8%],F:1.1%,M:1.8%,n:1614 |
|  | BR (SR/LR) | 27826 | 24960 | 89.70 | 0.96 | 5209 | 22617 | 0.23 | 4589 | 20363 | 88.10 | 90.03 | C:97.3%[S:90.9%,D:6.4%],F:0.8%,M:1.9%,n:1614 |
|  | TSB (SR/ST2) | 27178 | 25619 | 94.26 | 0.98 | 7515 | 19663 | 0.38 | 6569 | 19050 | 87.42 | 96.89 | C:98.6%[S:90.7%,D:7.9%],F:0.2%,M:1.2%,n:1614 |
|  | TSB (SR/TRINITY) | 27162 | 25546 | 94.05 | 0.98 | 7643 | 19519 | 0.39 | 6618 | 18923 | 86.56 | 96.95 | C:97.7%[S:90.9%,D:6.8%],F:0.9%,M:1.4%,n:1614 |
|  | TSB (LR/ST2) | 26373 | 24784 | 93.97 | 0.96 | 7245 | 19128 | 0.38 | 6245 | 18539 | 86.20 | 96.92 | C:96.8%[S:90.1%,D:6.7%],F:1.1%,M:2.1%,n:1614 |
|  | TSB (SR/LR/ST2) | 26545 | 24990 | 94.14 | 0.98 | 7313 | 19232 | 0.38 | 6329 | 18661 | 86.54 | 97.03 | C:98.1%[S:90.9%,D:7.2%],F:0.4%,M:1.5%,n:1614 |
|  | BR (RM2/SR) | 28660 | 25205 | 87.94 | 0.94 | 5176 | 23484 | 0.22 | 4557 | 20645 | 88.04 | 87.91 | C:97.3%[S:90.6%,D:6.7%],F:0.9%,M:1.8%,n:1614 |
|  | TSB (SR/RM2/ST2) | 27185 | 25505 | 93.82 | 0.97 | 7656 | 19529 | 0.39 | 6570 | 18935 | 85.81 | 96.96 | C:98.6%[S:91.5%,D:7.1%],F:0.3%,M:1.1%,n:1614 |
| ***Funaria*** | BR (SR) | 52000 | 22500 | 43.27 | 0.45 | 15089 | 36911 | 0.41 | 2737 | 19763 | 18.14 | 53.54 | C:85.8%[S:63.8%,D:22.0%],F:2.9%,M:11.3%,n:1614 |
|  | TSB (SR/ST2) | 45884 | 22608 | 49.27 | 0.64 | 25184 | 20700 | 1.22 | 4867 | 17201 | 19.32 | 83.10 | C:82.2%[S:69.3%,D:12.9%],F:2.8%,M:15.0%,n:1614 |
|  | TSB (SR/TRINITY) | 31928 | 16989 | 53.21 | 0.61 | 14603 | 17325 | 0.84 | 2724 | 14265 | 18.65 | 82.34 | C:86.6%[S:66.2%,D:20.4%],F:2.2%,M:11.2%,n:1614 |
|  | BR (SR/RM2+) | 50408 | 22142 | 43.92 | 0.59 | 14236 | 36175 | 0.39 | 2651 | 19491 | 18.62 | 53.88 | C:85.9%[S:64.7%,D:21.2%],F:2.4%,M:11.7%,n:1614 |
|  | TSB (SR/RM2+/ST2) | 45792 | 22013 | 48.07 | 0.61 | 26358 | 20688 | 1.27 | 4826 | 17187 | 18.31 | 83.08 | C:84.5%[S:41.2%,D:43.3%],F:3.0%,M:12.5%,n:1614 |
| ***Liriodendron*** | BR (SR) | 52157 | 31123 | 59.67 | 0.75 | 13404 | 38755 | 0.34 | 6707 | 24417 | 50.04 | 63.00 | C:90.8%[S:72.7%,D:18.1%],F:5.9%,M:3.3%,n:1614 |
|  | BR (LR) | 50343 | 30907 | 61.39 | 0.75 | 15568 | 34775 | 0.45 | 7618 | 23289 | 48.93 | 66.97 | C:88.2%[S:70.9%,D:17.3%],F:7.1%,M:4.7%,n:1614 |
|  | BR (SR/LR) | 51008 | 30709 | 60.20 | 0.75 | 12747 | 19996 | 0.64 | 6463 | 12556 | 50.70 | 62.79 | C:90.9%[S:72.1%,D:18.8%],F:5.3%,M:3.8%,n:1614 |
|  | TSB (SR/ST2) | 49150 | 33017 | 67.17 | 0.78 | 24180 | 24970 | 1.00 | 12145 | 20872 | 50.23 | 83.59 | C:84.2%[S:73.5%,D:10.7%],F:8.2%,M:7.6%,n:1614 |
|  | TSB (SR/TRINITY) | 52407 | 34427 | 65.70 | 0.77 | 26528 | 25879 | 1.02 | 13196 | 21231 | 49.74 | 82.04 | C:84.7%[S:74.2%,D:10.5%],F:7.6%,M:7.7%,n:1614 |
|  | TSB (LR/ST2) | 49740 | 33166 | 66.68 | 0.78 | 25137 | 24603 | 1.02 | 12727 | 20439 | 50.63 | 83.08 | C:82.9%[S:72.5%,D:10.4%],F:9.0%,M:8.1%,n:1614 |
|  | BR (SR/RM2+) | 51788 | 31277 | 60.39 | 0.76 | 13566 | 38222 | 0.35 | 6806 | 24469 | 50.17 | 64.02 | C:88.9%[S:71.4%,D:17.5%],F:6.3%,M:4.8%,n:1614 |
|  | TSB (SR/LR/ST2) | 51630 | 34301 | 66.44 | 0.78 | 26198 | 25432 | 1.03 | 13205 | 21096 | 50.40 | 82.95 | C:83.2%[S:72.4%,D:10.8%],F:9.4%,M:7.4%,n:161 |
|  | TSB (SR/RM2+/ST2) | 50666 | 34096 | 67.29 | 0.78 | 25815 | 24851 | 1.04 | 13320 | 20776 | 51.60 | 83.60 | C:83.6%[S:72.9%,D:10.7%],F:9.1%,M:7.3%,n:1614 |
| ***Populus*** | BR (SR) | 48424 | 36568 | 75.52 | 0.87 | 9434 | 38991 | 0.24 | 6666 | 29902 | 70.66 | 76.69 | C:97.9%[S:75.0%,D:22.9%],F:1.0%,M:1.1%,n:1614 |
|  | BR (LR) | 47104 | 35851 | 76.11 | 0.87 | 10513 | 36606 | 0.29 | 7281 | 28569 | 69.26 | 78.04 | C:96.4%[S:76.0%,D:20.4%],F:2.1%,M:1.5%,n:1614 |
|  | BR (SR/LR) | 48715 | 36523 | 74.97 | 0.86 | 10529 | 38188 | 0.27 | 6790 | 29733 | 64.49 | 77.86 | C:97.8%[S:75.0%,D:22.8%],F:0.9%,M:1.3%,n:1614 |
|  | TSB (SR/ST2) | 40841 | 35948 | 88.02 | 0.94 | 13068 | 27773 | 0.47 | 9354 | 26594 | 71.58 | 95.75 | C:97.5%[S:72.2%,D:25.3%],F:0.7%,M:1.8%,n:1614 |
|  | TSB (SR/TRINITY) | 38517 | 33686 | 87.46 | 0.93 | 12621 | 25896 | 0.49 | 8814 | 24872 | 69.83 | 96.04 | C:96.5%[S:73.3%,D:23.2%],F:1.1%,M:2.4%,n:1614 |
|  | TSB (LR/ST2) | 38913 | 34061 | 87.53 | 0.93 | 12928 | 25985 | 0.50 | 9095 | 24965 | 70.35 | 96.07 | C:94.9%[S:73.2%,D:21.7%],F:2.2%,M:2.9%,n:1614 |
|  | TSB (SR/LR/ST2) | 40758 | 35931 | 88.16 | 0.94 | 13014 | 27744 | 0.47 | 9336 | 26595 | 71.74 | 95.86 | C:96.1%[S:72.1%,D:24.0%],F:1.7%,M:2.2%,n:1614 |
|  | BR (SR/RM2 +) | 47956 | 36528 | 76.17 | 0.87 | 10494 | 37461 | 0.28 | 6717 | 29803 | 64.01 | 79.56 | C:98.0%[S:74.8%,D:23.2%],F:0.8%,M:1.2%,n:1614 |
|  | BR (SR/RM2 +/ST2) | 42093 | 36016 | 85.56 | 0.92 | 14161 | 27932 | 0.51 | 9366 | 26650 | 66.14 | 95.41 | C:96.5%[S:73.4%,D:23.1%],F:1.7%,M:1.8%,n:1614 |
| ***Rosa*** | BR (SR) | 47318 | 32248 | 68.15 | 0.81 | 12944 | 34370 | 0.38 | 7275 | 24970 | 56.20 | 72.65 | C:96.4%[S:86.8%,D:9.6%],F:1.9%,M:1.7%,n:1614 |
|  | BR (LR) | 47411 | 26290 | 55.45 | 0.81 | 13407 | 34006 | 0.39 | 6429 | 19861 | 47.95 | 58.40 | C:82.9%[S:79.4%,D:3.5%],F:1.9%,M:15.2%,n:1614 |
|  | BR (SR/LR) | 49128 | 33002 | 67.17 | 0.80 | 14077 | 35046 | 0.40 | 7642 | 25356 | 54.29 | 72.35 | C:96.9%[S:86.9%,D:10.0%],F:1.6%,M:1.5%,n:1614 |
|  | TSB (SR/ST2) | 44577 | 33814 | 75.85 | 0.85 | 19147 | 25430 | 0.75 | 11144 | 22670 | 58.20 | 89.15 | C:98.0%[S:86.4%,D:11.6%],F:0.4%,M:1.6%,n:1614 |
|  | TSB (SR/TRINITY) | 45971 | 34424 | 74.88 | 0.84 | 19923 | 26048 | 0.76 | 11384 | 23040 | 57.14 | 88.45 | C:97.6%[S:86.1%,D:11.5%],F:0.9%,M:1.5%,n:1614 |
|  | TSB (LR/ST2 | 44845 | 34071 | 75.97 | 0.85 | 19241 | 25604 | 0.75 | 11349 | 22722 | 58.98 | 88.74 | C:96.6%[S:83.8%,D:12.8%],F:1.1%,M:2.3%,n:1614 |
|  | TSB (SR/LR/ST2) | 48675 | 35380 | 72.69 | 0.83 | 21593 | 27082 | 0.80 | 11833 | 23547 | 54.80 | 86.95 | C:97.9%[S:85.3%,D:12.6%],F:0.5%,M:1.6%,n:1614 |
