# Supplementary material for: Welcome to the big leaves: Best practices for improving genome annotation in non‐model plant genomes
Source: Appl Plant Sci. 2023 Aug 8;11(4):e11533. doi: 10.1002/aps3.11533 (PMC10439824; doi:10.1002/aps3.11533)
Supplement: Supplementary file 11 — Appendix S11. Liriodendron masked and unmasked. [file APS3-11-e11533-s011.docx]

**Appendix S11.** *Liriodendron* masked and unmasked.

| **Masking** | **Mono** | **Multi** | **Input format** |
| --- | --- | --- | --- |
| Unmasked | 59021 | 148473 | BR (SR) |
|  | 45299 | 133575 | BR (SR/LR) |
|  | 67597 | 127324 | BR (LR) |
| Repeat masked | 13403 | 38752 | BR (SR) |
|  | 12748 | 38259 | BR (SR/LR) |
|  | 15568 | 34774 | BR (LR) |
| Additional masking for LTRs | 13566 | 38222 | BR (SR/RM2+) |
