# Supplementary material for: Welcome to the big leaves: Best practices for improving genome annotation in non‐model plant genomes
Source: Appl Plant Sci. 2023 Aug 8;11(4):e11533. doi: 10.1002/aps3.11533 (PMC10439824; doi:10.1002/aps3.11533)
Supplement: Supplementary file 12 — Appendix S12. Comparison between StringTie2 and BRAKER runs. [file APS3-11-e11533-s010.docx]

**Appendix S12.** Comparison between StringTie2 and BRAKER runs.

| **Stringtie** | | | | | | | | **BRAKER** | | | | | |
| --- | --- | --- | --- | --- | --- | --- | --- | --- | --- | --- | --- | --- | --- |
| **Species** | **Run** | **mono** | **multi** | **total** | **ratio** | **BUSCO** | **EnTAP annoation** | **mono** | **multi** | **total** | **ratio** | **BUSCO** | **EnTAP annoation** |
| ***Arabidopsis*** | SR | 6236 | 29287 | 35523 | 0.21 | C:95.5%[S:70.7%,D:24.8%],F:1.1%,M:3.4%,n:1614 | 89.13 | 7515 | 19663 | 27178 | 0.38 | C:95.9%[S:88.2%,D:7.7%],F:0.2%,M:3.9%,n:1614 | 89.87 |
|  | LR | 6062 | 30117 | 36179 | 0.20 | C:84.7%[S:61.2%,D:23.5%],F:4.9%,M:10.4%,n:1614 | 83.77 | 7245 | 19128 | 26373 | 0.3787641154 | C:93.9%[S:87.5%,D:6.4%],F:1.1%,M:5.0%,n:1614 | 89.61 |
|  | SR+LR | 7266 | 34885 | 42151 | 0.21 | C:93.6%[S:65.1%,D:28.5%],F:0.9%,M:5.5%,n:1614 | 84.52 | 7313 | 19232 | 26545 | 0.3802516639 | C:95.6%[S:88.5%,D:7.1%],F:0.4%,M:4.0%,n:1614 | 89.70 |
|  | SR (RM2+) | 6193 | 29287 | 35480 | 0.21 | C:95.5%[S:71.0%,D:24.5%],F:1.1%,M:3.4%,n:1614 | 89.14 | 7656 | 19529 | 27185 | 0.3920323621 | C:95.7%[S:88.8%,D:6.9%],F:0.3%,M:4.0%,n:1614 | 87.94 |
| ***Funaria*** | SR | 13166 | 45543 | 58709 | 0.29 | C:84.5%[S:41.2%,D:43.3%],F:3.0%,M:12.5%,n:1614 | 70.00 | 25184 | 20700 | 45884 | 1.22 | C:84.9%[S:65.1%,D:19.8%],F:2.2%,M:12.9%,n:1614 | 43.27 |
|  | SR (RM2+) | 13166 | 45543 | 58709 | 0.29 | C:84.5%[S:41.2%,D:43.3%],F:3.0%,M:12.5%,n:1614 | 70.00 | 26358 | 20688 | 47046 | 1.274071926 | C:84.7%[S:65.1%,D:19.6%],F:2.2%,M:13.1%,n:1614 | 43.93 |
| ***Populus*** | SR | 10799 | 38162 | 48961 | 0.28 | C:73.3%[S:45.0%,D:28.3%],F:6.9%,M:19.8%,n:1614 | 82.70 | 13068 | 27773 | 40841 | 0.47 | C:95.6%[S:70.9%,D:24.7%],F:0.8%,M:3.6%,n:1614 | 75.52 |
|  | LR | 2744 | 17873 | 20617 | 0.15 | C:51.6%[S:42.1%,D:9.5%],F:12.7%,M:35.7%,n:1614 | 91.17 | 12928 | 25985 | 38913 | 0.50 | C:93.3%[S:72.2%,D:21.1%],F:1.9%,M:4.8%,n:1614 | 76.11 |
|  | SR +LR | 3322 | 11067 | 14389 | 0.30 | C:40.3%[S:33.5%,D:6.8%],F:5.3%,M:54.4%,n:1614 | 87.46 | 13014 | 27744 | 40758 | 0.47 | C:94.5%[S:71.0%,D:23.5%],F:1.6%,M:3.9%,n:1614 | 74.97 |
|  | SR (RM2+) | 10799 | 38166 | 48965 | 0.28 | C:73.3%[S:45.0%,D:28.3%],F:6.9%,M:19.8%,n:1614 | 82.74 | 14161 | 27932 | 42093 | 0.5069812402 | C:94.6%[S:72.0%,D:22.6%],F:1.7%,M:3.7%,n:1614 | 76.17 |
| ***Liriodendron*** | SR | 16793 | 44714 | 61507 | 0.37 | C:87.1%[S:52.0%,D:35.1%],F:7.2%,M:5.7%,n:1614 | 70.33 | 24180 | 24970 | 49150 | 0.9683620344 | C:81.1%[S:70.9%,D:10.2%],F:7.9%,M:11.0%,n:1614 | 59.67 |
|  | LR | 11755 | 21887 | 33642 | 0.54 | C:65.7%[S:52.4%,D:13.3%],F:13.1%,M:21.2%,n:1614 | 71.39 | 25137 | 24603 | 49740 | 1.02170467 | C:79.5%[S:69.8%,D:9.7%],F:8.2%,M:12.3%,n:1614 | 61.40 |
|  | SR +LR | 16792 | 44716 | 61508 | 0.37 | C:77.3%[S:58.5%,D:18.8%],F:13.3%,M:9.4%,n:1614 | 70.240141 | 26198 | 25432 | 51630 | 1.03 | C:80.1%[S:69.8%,D:10.3%],F:8.8%,M:11.1%,n:1614 | 60.20 |
|  | SR (RM2+) | 17018 | 40257 | 57275 | 0.42 | C:75.2%[S:45.2%,D:30.0%],F:10.0%,M:14.8%,n:1614 | 67.84 | 25815 | 24851 | 50666 | 1.038791196 | C:80.8%[S:70.6%,D:10.2%],F:8.4%,M:10.8%,n:1614 | 60.40 |
| ***Rosa*** | SR | 13382 | 39857 | 53239 | 0.33 | C:97.0%[S:68.2%,D:28.8%],F:0.7%,M:2.3%,n:1614 | 78.81 | 19147 | 25430 | 44577 | 0.7529296107 | C:95.8%[S:84.0%,D:11.8%],F:0.4%,M:3.8%,n:1614 | 68.15 |
|  | LR | 19582 | 54372 | 73954 | 0.36 | C:88.7%[S:57.0%,D:31.7%],F:4.4%,M:6.9%,n:1614 | 71.97 | 19241 | 25604 | 44845 | 0.75 | C:94.4%[S:81.5%,D:12.9%],F:1.2%,M:4.4%,n:1614 | 55.45 |
|  | SR +LR | 28178 | 76552 | 104730 | 0.37 | C:97.2%[S:49.9%,D:47.3%],F:0.8%,M:2.0%,n:1614 | 71.97 | 21593 | 27082 | 48675 | 0.7973192526 | C:95.8%[S:83.3%,D:12.5%],F:0.7%,M:3.5%,n:1614 | 67.17 |
