# Supplementary material for: Welcome to the big leaves: Best practices for improving genome annotation in non‐model plant genomes
Source: Appl Plant Sci. 2023 Aug 8;11(4):e11533. doi: 10.1002/aps3.11533 (PMC10439824; doi:10.1002/aps3.11533)
Supplement: Supplementary file 13 — Appendix S13. Overlaps between BR (SR) and BR (SR/ST2) in Liriodendron. [file APS3-11-e11533-s009.docx]

**Appendix S13.** Overlaps between BR (SR) and BR (SR/ST2) in *Liriodendron*.

| **Run** | **Mono** | **Multi** | **Overlap with BR (SR)** |
| --- | --- | --- | --- |
| **BR (SR)** | 13404 | 38755 |  |
| **TSB (SR/ST2)** | 24180 | 24970 | 15234 |
| **TSB (SR/OrthoDB)** | 23190 | 23518 | 11111 |
