# Supplementary material for: Welcome to the big leaves: Best practices for improving genome annotation in non‐model plant genomes
Source: Appl Plant Sci. 2023 Aug 8;11(4):e11533. doi: 10.1002/aps3.11533 (PMC10439824; doi:10.1002/aps3.11533)

**Appendix S14.** Genes predicted as mono-exonic from the BR(SR/ST2) run overlapping with multis from the BR(SR) run in *Liriodendron*.


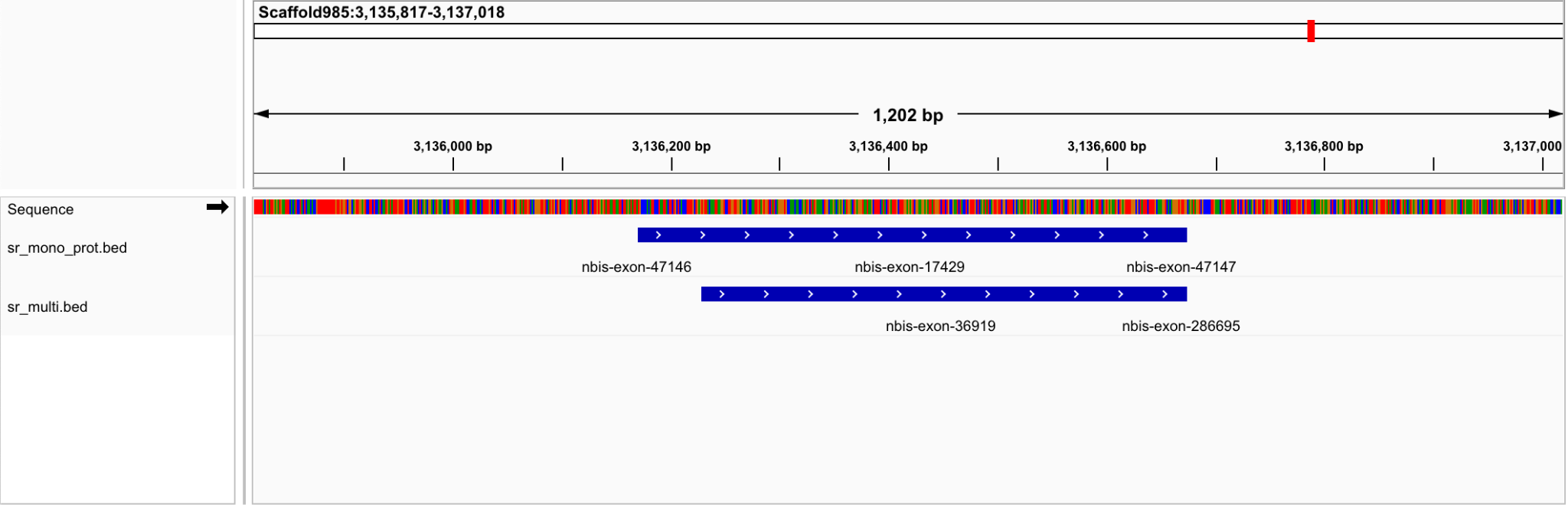

Supplement: Supplementary file 14 — Appendix S14. Genes predicted as mono‐exonic from the BR (SR/ST2) run overlapping with multis from the BR (SR) run in Liriodendron. [file APS3-11-e11533-s007.docx]
