# Supplementary material for: Welcome to the big leaves: Best practices for improving genome annotation in non‐model plant genomes
Source: Appl Plant Sci. 2023 Aug 8;11(4):e11533. doi: 10.1002/aps3.11533 (PMC10439824; doi:10.1002/aps3.11533)
Supplement: Supplementary file 15 — Appendix S15. Comparison between MAKER and BRAKER. [file APS3-11-e11533-s008.docx]

**Appendix S15.** Comparison between MAKER and BRAKER.

|  | **MAKER (RM2+)** | | | | | **BR (SR)** | | | | |
| --- | --- | --- | --- | --- | --- | --- | --- | --- | --- | --- |
| **Species** | **BUSCO** | **gFACS** | | **canonical splice (%)** | **Gene lengths** | **BUSCO** | **gFACS** | | **canonical splice (%)** | **Gene lengths** |
|  |  | **monoexonic** | **multiexonic** |  |  |  | **monoexonic** | **multiexonic** |  |  |
| ***Arabidopsis thaliana*** | C:90.4%[S:89.0%,D:1.4%],F:3.2%,M:6.4%,n:1614 | 4152 | 18341 | 99.82 | 2342.81 | C:96.3%[S:90.2%,D:6.1%],F:1.0%,M:2.7%,n:1614 | 5066 | 22300 | 99.06 | 2936.07 |
| ***Populus trichocarpa*** | C:19.6%[S:17.9%,D:1.7%],F:1.5%,M:78.9%,n:1614 | 330 | 7273 | 98.39 | 6135.47 | C:97.9%[S:75.0%,D:22.9%],F:1.0%,M:1.1%,n:1614 | 9434 | 38991 | 98.75 | 3142.98 |
| ***Funaria hygrometrica*** | C:79.6%[S:66.7%,D:12.9%],F:4.9%,M:15.5%,n:1614 | 18930 | 25468 | 99.86 | 1868.69 | C:85.8%[S:63.8%,D:22.0%],F:2.9%,M:11.3%,n:1614 | 15084 | 36909 | 99.04 | 2112.89 |
